# Advances in Cell and Immune Therapies for Melanoma

**DOI:** 10.3390/biomedicines13010098

**Published:** 2025-01-03

**Authors:** Tanase Timis, Sanda Buruiana, Delia Dima, Madalina Nistor, Ximena Maria Muresan, Diana Cenariu, Adrian-Bogdan Tigu, Ciprian Tomuleasa

**Affiliations:** 1 Department of Hematology, Iuliu Hațieganu University of Medicine and Pharmacy, 400347 Cluj-Napoca, Romania; 2 Department of Oncology, Bistrița Emergency Hospital, 420094 Bistrița, Romania; 3 Department of Hematology, Nicolae Testemițanu University of Medicine and Pharmacy, MD-2004 Chisinau, Moldova; 4 Department of Hematology, Ion Chiricuta Oncology Institute, 400015 Cluj-Napoca, Romania; 5 Department of Personalized Medicine and Rare Diseases, MEDFUTURE—Institute for Biomedical Research, “Iuliu Hațieganu” University of Medicine and Pharmacy, 400349 Cluj-Napoca, Romania

**Keywords:** melanoma, immune therapy, cell therapy, treatment resistance

## Abstract

The incidence rate of cutaneous melanoma is on the rise worldwide, due to increased exposure to UV radiation, aging populations, and exposure to teratogen agents. However, diagnosis is more precise, and the increased number of new cases is related to the improved diagnosis tools. Despite better early diagnosis and better therapies, melanoma has remained a significant public health challenge because of its aggressive behavior and high potential for metastasis. In 2020, cutaneous melanoma constituted approximately 1.3% of all cancer deaths that occurred within the European Union, thereby highlighting the necessity for effective prevention, timely diagnosis, and sustainable treatment measures, especially as a growing number of cases occur among younger patients. Melanoma is regarded as one of the most inflamed cancers due to its high immune cell presence and strong response to immunotherapy, fueling the need for development of immune-driven innovative treatments. Approved therapies, including immune checkpoint inhibitors (e.g., anti-PD-1 and anti-CTLA-4), have notably improved survival rates in melanoma. However, the limitations of the PD-1/PD-L1 and CTLA-4 axes inhibitors, such as low response rates, treatment resistance, and toxicity, have driven the need for continued research and advancements in treatment strategies. Current clinical trials are exploring various combinations of immune checkpoint inhibitors with costimulatory receptor agonists, chemotherapy, targeted therapies, and other immunotherapies, with the goal of improving outcomes and reducing side effects for melanoma patients. Emerging approaches, including adoptive cell therapy with tumor-infiltrating lymphocytes (TILs) and oncolytic virotherapy, are showing promise. While CAR-T cell therapy has been less successful in melanoma compared to blood cancers, ongoing research is addressing challenges like the tumor microenvironment and antigen specificity. This review provides an overview of the requirement for advances in these medications, to mark a significant step forward in melanoma management, set to bring a fresh breath of hope for patients.

## 1. Introduction

Melanoma represents a unique model for studying cancer immunology due to its complex interactions with the immune system. The interplay between genetic mutations, environmental factors, and the immune response is crucial for determining treatment outcomes. Approximately 30–40% of all melanoma subtypes have a high tumor mutational burden (TMB). Studies indicate that tumors with elevated TMB are often more responsive to immunotherapy [1]. For example, desmoplastic melanomas exhibit a high TMB, correlating with a favorable response to immune checkpoint inhibitors (ICIs) [2]. Clinical evidence suggests that the response rate for desmoplastic melanoma treated with ICIs typically exceeds 70% [3].

**Figure 1:**
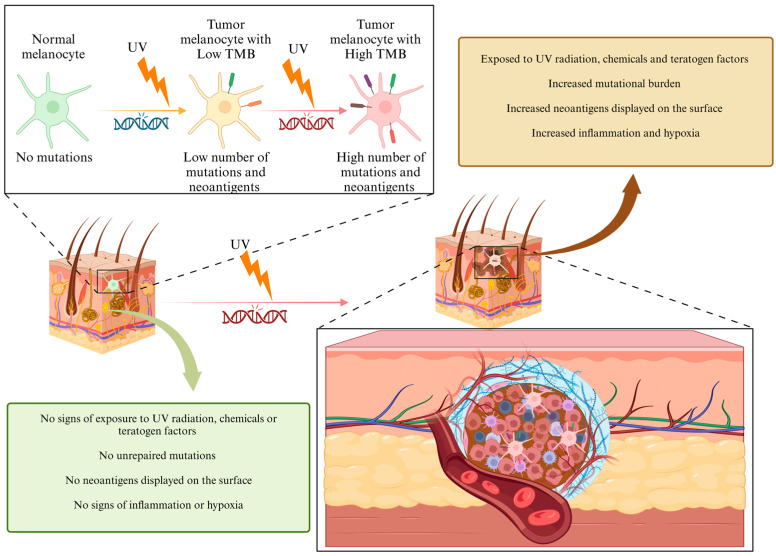
Melanocytes shift from normal to tumor type. The UV radiation and other mutagen factors generate skin damage and induce mutations in the melanocytes’ DNA. The poor efficacy of the DNA repair mechanism allows mutation accumulation in the DNA and at high tumor mutation burden (TMB) the tumor melanocytes express neoantigens. The tumor site is prone to becoming hypoxic and new blood vessels are created which increases the proinflammatory state in the tumor microenvironment (TME) and promotes tumor cells escape and metastasis. Figure created with Biorender.

High TMB primarily results from ultraviolet (UV) radiation exposure [2]. UV radiation is a potent mutagen, and its role in melanoma is well-documented (Figure 1). It induces specific types of DNA damage, such as cyclobutene pyrimidine dimers and 6–4 photoproducts, which, if not adequately repaired, lead to mutations during DNA replication [4,5,6]. The accumulation of these mutations results in the formation of neoantigens—peptides derived from mutated proteins that are not present in normal tissues. Neoantigens can serve as critical targets for T cells, as they elicit a robust immune response, distinguishing tumor cells from healthy cells [7].

Schumacher et al. demonstrated that the presence of neoantigens correlates with T cell infiltration and responses to ICIs in melanoma [8]. The study emphasized that high TMB increases the probability of neoantigen formation, which can enhance the recognition and elimination of tumor cells by the immune system. Research conducted by Hugo et al. revealed that the mutation load and the resulting neoantigens are strong predictors of the response to anti-PD-1 therapy in melanoma patients [9]. The discrepancy in tumor location between *BRAF*- and *NRAS*-mutated melanomas can be attributed to the differing sensitivity of *BRAF*- and *NRAS*-mutant melanocytes to ultraviolet (UV) light-induced carcinogenesis, with BRAF-mutated melanocytes exhibiting greater sensitivity to UV-mediated damage [10].

However, not all neoantigens are equally immunogenic. The effectiveness of neoantigens in eliciting T cell responses depends on their ability to be properly presented by major histocompatibility complex (MHC) molecules. Mutations in genes involved in antigen processing and presentation, such as *B2M* and *HLA*, can lead to downregulation of MHC molecules, significantly impairing the ability of the immune system to recognize and attack melanoma cells [11].

The relationship between TMB and clinical outcomes is complex (Table 1), as various components of the tumor microenvironment (TME), such as cancer-associated fibroblasts, lymphocytes, myeloid-derived suppressor cells, tumor-associated macrophages, dendritic cells, and extracellular matrix, play crucial roles [12].

The TME in melanoma is characterized by a dynamic and immunosuppressive environment that evolves as the tumor progresses. Initially, the immune system effectively identifies and eliminates melanoma cells; however, the remaining melanoma cells adapt and escape immune surveillance through a process known as immunoediting [13,14]. This phenomenon involves the selection of variants that can evade immune detection, leading to treatment resistance. Melanoma cells often exploit immune checkpoints such as PD-1/PD-L1 and CTLA-4, which serve as crucial modulators of T cells activity. These checkpoints allow melanoma cells to inhibit T cell activation and proliferation, facilitating their escape from immune surveillance [15,16,17]. Other studies demonstrated that melanoma cells could upregulate PD-L1 in response to interferon-gamma (IFN-γ) released by activated T cells, effectively dampening the immune response [15,18].

Within the TME, the function of dendritic cells (DCs) can be impaired. These cells are crucial for presenting antigens to T cells; however, in melanoma, their ability to effectively activate T cells may be diminished due to local immunosuppressive factors [19]. Additionally, the presence of immunosuppressive cell populations, including regulatory T cells (Tregs) and myeloid-derived suppressor cells (MDSCs), further complicates the immune landscape. Research has shown that higher levels of Tregs within the TME correlate with poorer prognosis in melanoma patients, indicating their role in promoting immune tolerance [20]. Genetic and epigenetic alterations also significantly influence melanoma’s development and response to therapy. Moreover, epigenetic modifications may silence the expression of neoantigens or alter immune signaling pathways, contributing to immune escape mechanisms [21].

The inflammatory secretome within the TME also plays a crucial role in shaping immune responses. Cytokines, such as IL-6 and IL-10, promote the expansion and activity of Tregs and MDSCs, thereby fostering an immunosuppressive environment that supports tumor growth and enhances resistance to therapies [22]. Studies have shown that melanoma cells can release these cytokines to create an inflammatory milieu that further complicates immune responses [23]. As melanoma progresses, the TME becomes increasingly immunosuppressive, particularly at the tumor–stromal interface. The establishment of cytokine gradients and the expression of immune checkpoint ligands like PD-L1 on macrophages and DCs contribute to this suppressive environment [24]. Invasive melanoma tumors are adept of creating consolidated suppressive niches, allowing them to evade immune detection even in the presence of cytotoxic T cells [9].

As an initial conclusion, it is clear that anti-PD-1 (e.g., pembrolizumab, nivolumab) and anti-CTLA-4 (e.g., ipilimumab) checkpoint inhibitors have significant efficacy in treating melanoma, and it has been demonstrated that increased TMB [25], together with mutations in driver oncogenes, such as *BRAF* and *NRAS*, generate neoantigens that enhance immunogenicity, leading to stronger immune responses to specific inhibitors [26]. However, melanomas are capable of developing various immune evasion strategies—including TME-induced suppression, genetic alterations, and metabolic shifts—which, in the end, all contribute to treatment resistance and tumor relapse [27].

**Table 1:** Key immune checkpoint inhibitors approved in melanoma.

Target	Drug	Approval Year	Therapy Strategy	Indications	Clinical Trial ID	Key Clinical Findings	Limitations	Ref.
Anti-CTLA-4	Ipilimumab	2011	Monotherapy	- Advanced/metastatic melanoma	NCT00094653	- OS improvement: 10.1 months (vs. 6.4 months for gp100 vaccine).	- High toxicity profile (immune related).	[28]
Anti-PD-1	Nivolumab	2014	Monotherapy	- Advanced/metastatic melanoma	NCT01721772	- OS at 1 year: ~72%. \n- ORR: 40% (treatment-naïve).	Focused on wild-type BRAF populations.	[29]
Anti-PD-1	Pembrolizumab	2014	Monotherapy	- Advanced/metastatic melanoma	NCT01866319	- PFS: ~5.5 months (vs. 2.8 months with ipilimumab).	No head-to-head OS benefit with nivolumab.	[30]
Anti-PD-1 + Anti-CTLA-4	Nivolumab + Ipilimumab	2015	Combination therapy	- Advanced/metastatic melanoma	NCT01844505	- OS at 3 years: ~58% (combo) vs. 43% (ipi alone).	Increased toxicity compared to monotherapy.	[31]
Anti-PD-1 + Anti-LAG-3	Relatlimab + Nivolumab	2022	Combination therapy	- Advanced/metastatic melanoma (treatment-naïve)	NCT03470922	- PFS: ~10.1 months (combo) vs. 4.6 months (nivolumab alone).	- OS data not yet available.	[32]

## 2. Alternative Immune Checkpoints Inhibitors and Co-Stimulatory Receptors’ Agonists

Despite the success of CTLA-4 and PD-1 inhibitors, a significant percentage of patients either do not respond to or develop resistance to these therapies. This has spurred research into alternative immune checkpoints to overcome immune escape mechanisms.

LAG-3 (Lymphocyte Activation Gene-3) is an inhibitory receptor expressed on exhausted T cells and Tregs, where it suppresses immune responses by binding to MHC class II molecules. LAG-3 competes with CD4 for MHC class II binding, inhibiting therefore T cell activation and proliferation. The Relativity-047 trial is a first-line study evaluating the efficacy and safety of the combination of relatlimab (anti-LAG-3) and nivolumab (anti-PD-1) in advanced melanoma. Current findings suggest that this combination demonstrates efficacy comparable to the established ipilimumab (anti-CTLA-4) and nivolumab (ipi/nivo) combination in this setting. However, Relativity-047 appears to offer a more favorable toxicity profile, addressing one of the significant concerns with ipi/nivo therapy. Despite these promising results, overall survival (OS) data for the relatlimab/nivolumab combination remain immature, necessitating further follow-up to establish the long-term benefits. In the second-line or pre-treated patient population, findings from the Relativity-020 trial suggest that the relatlimab/nivolumab combination demonstrates lower activity compared to ipilimumab/nivolumab in this setting. Given these observations, the relatlimab/nivolumab combination is currently a viable consideration for first-line treatment, while awaiting mature OS data to confirm its long-term efficacy compared to the ipilimumab/nivolumab standard of care [33].

TIM-3 (T cell immunoglobulin and mucin domain-3) is another inhibitory receptor implicated in T cell exhaustion, particularly in patients who become resistant to PD-1 blockade. TIM-3 is expressed in various immune cells, including T cells and dendritic cells, and its engagement inhibits Th1 cell responses and promotes T cell apoptosis. Preclinical studies have shown that co-blockade of TIM-3 and PD-1 can restore T cell function and enhance anti-tumor responses [34]. Several clinical trials are currently evaluating anti-TIM-3 therapies, like sabatolimab (anti-TIM-3), in combination with spartalizumab (anti-PD-1) in advanced melanoma [35], indicating its potential as a critical target for overcoming resistance mechanisms.

TIGIT (T cell immunoreceptor with Ig and ITIM domains) competes with the co-stimulatory receptor CD226 for binding to CD155, which is expressed in tumor cells and antigen-presenting cells. By inhibiting CD226 signaling, TIGIT suppresses T cell and natural killer (NK) cell functions, contributing to the tumor’s immune evasion. In a preclinical study, TIGIT blockade combined with PD-1 inhibition enhanced anti-tumor responses in melanoma models [36]. Ongoing clinical trials are evaluating anti-TIGIT antibodies in melanoma patients, particularly those with high TIGIT expression, to determine their effectiveness in enhancing anti-tumor immune responses [37].

VISTA (V-domain Ig Suppressor of T cell Activation) primarily regulates immune tolerance within the TME, through its impact on myeloid cells. VISTA is expressed on a range of immune cells, including MDSCs and Tregs, and it is upregulated following PD-1 inhibition, making it a promising target for patients who develop resistance to PD-1 therapy [38]. Research suggests that VISTA blockade can reprogram tumor-associated macrophages and enhance CD8+ T cell activity, offering a new approach to overcoming immune suppression within the TME [39]. This highlights the importance of understanding the tumor microenvironment in developing effective combination strategies that can enhance anti-tumor immunity by targeting multiple inhibitory pathways simultaneously.

The immune response, particularly T cell activation, is governed by a delicate balance between inhibitory and stimulatory signals. Co-stimulatory receptors on T cells provide the essential secondary signals needed for full T cell activation and sustained anti-tumor responses, complementing antigen recognition by the T cell receptor (TCR). While immune checkpoint inhibitors (like anti-CTLA-4 and anti-PD-1) block inhibitory signals, agonists targeting co-stimulatory receptors enhance the immune response by activating T cells, boosting their proliferation, survival, and effector functions (Table 2). These co-stimulatory pathways are crucial for optimizing immune responses, particularly in patients resistant to checkpoint blockade.

OX40 (CD134) is a member of the TNF receptor superfamily, expressed in activated T cells, Tregs, and NK cells. The binding of OX40 to its ligand (OX40L) promotes T cell proliferation, survival, and memory T cell generation, while reducing Treg-mediated suppression. OX40 agonists enhance the cytotoxic function of CD8+ T cells and reduce Treg activity, which is beneficial in melanoma where regulatory T cell suppression represents a significant barrier. In early-phase trials, OX40 agonists have been tested in combination with checkpoint inhibitors, showing enhanced responses compared to monotherapy [40].

CD137 (4-1BB) is another TNF receptor family member that is upregulated on activated CD8+ T cells, NK cells, and dendritic cells (DCs). Activation of CD137 enhances T cell proliferation, promotes survival through anti-apoptotic pathways, and increases the cytotoxic activity of both CD8+ T cells and NK cells. Preclinical and clinical trials demonstrate that CD137 agonists can potentiate the effects of PD-1 blockade by enhancing both T cell- and NK cell-mediated responses against melanoma. Combining CD137 agonists with PD-1 inhibitors has shown synergistic effects, improving anti-tumor responses in melanoma models [41,42].

GITR (Glucocorticoid-Induced TNFR-related protein) is expressed in both effector T cells and Tregs. Upon activation, GITR signaling enhances effector T cell proliferation and function, while reducing the suppressive activity of Tregs. This dual action of promoting effector T cell activity and inhibiting Treg function makes GITR a promising therapeutic target. GITR agonists have demonstrated preclinical efficacy in enhancing T cell responses and reducing Treg-mediated immune suppression. In melanoma, early-phase trials have shown that GITR agonists can boost the anti-tumor activity of checkpoint inhibitors, although robustness of clinical benefits is still under investigation [43].

ICOS (Inducible T cell Co-Stimulator) is expressed in activated T cells and plays a critical role in sustaining T cell activation, proliferation, and cytokine production. The ICOS pathway is essential for promoting the development and function of follicular helper T cells, which support B-cell-mediated immunity. ICOS agonists have shown potential in enhancing anti-tumor immunity, particularly in combination with CTLA-4 inhibitors. For example, ipilimumab treatment has been associated with increased ICOS expression on T cells, suggesting that ICOS signaling may enhance the efficacy of CTLA-4 blockade [44]. Ongoing trials are investigating the use of ICOS agonists in melanoma, particularly in patients with suboptimal responses to checkpoint inhibitors.

CD40 is a co-stimulatory receptor found primarily in antigen-presenting cells (APCs), such as dendritic cells, macrophages, and B cells. Activation of CD40 enhances the ability of APCs to present antigens, secrete cytokines, and prime T cell responses. CD40 signaling bridges innate and adaptive immunity, making it a powerful target for cancer immunotherapy. CD40 agonists are being investigated for their ability to activate APCs and improve the efficacy of T cell-based therapies. Preclinical studies have shown that combining CD40 agonists with checkpoint inhibitors or chemotherapy can enhance anti-tumor immunity in melanoma [41,45].

**Figure 2:**
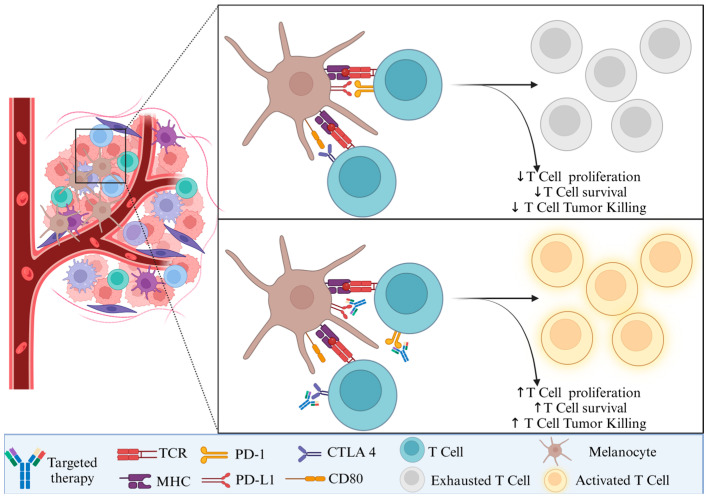
Targeted therapies anti PD-1, anti PD-L1 and anti CTLA-4 (CD152) in melanoma. In the TME, the malign melanocytes interact with T cells, and the MHC binds to the antigen and TCR. In the first situation, when PD-1 binds to PD-L1, and CTLA 4 binds to CD80, the T cells will exhaust, will slow in proliferation, and will lose their killing ability. On the other hand, when antibodies designed to target PD-1, PD-L1 or CTLA 4 interact with the target, T cell activity is restored and their proliferation is stimulated, as well as their tumor killing ability.

Combination strategies using co-stimulatory receptor agonists are particularly promising in combination with other immunotherapies, such as checkpoint inhibitors (e.g., anti-PD-1, anti-CTLA-4), cytokine therapies, and tumor vaccines. These combinations aim to (i) enhance T cell priming and expansion by amplifying the initial activation signals provided by APCs, improving the efficacy of vaccines and TIL-based therapies, (ii) boost effector T cell function to overcome T cell exhaustion, restoring the ability of T cells to kill cancer cells, and (iii) reduce immune suppression by targeting receptors like OX40 and GITR to diminish the suppressive effects of Tregs within the tumor microenvironment. For example, a phase I clinical trial investigating OX40 agonists in combination with checkpoint inhibitors showed durable responses in some patients, highlighting the potential for these agents to improve outcomes in melanoma [40]. Similarly, CD137 agonists combined with PD-1 inhibitors have shown synergy in preclinical models and early-phase clinical trials [41]. Targeting the neoantigens, as well as PD-1 or CTLA 4, may result in T cell activation and increased antitumor activity (Figure 2).

However, challenges remain in the use of co-stimulatory agonists, as they can lead to immune-related adverse events (irAEs) and toxicity due to excessive immune activation. Hepatotoxicity, colitis, and cytokine release syndrome are among the common toxicities observed with these agents, making it crucial to balance efficacy and safety. Additionally, optimal dosing and timing of co-stimulatory receptor agonists in combination with other therapies are critical to maximizing their benefits while minimizing toxicities. Preclinical models enforce that the timing of administration can significantly influence therapeutic outcomes, as delayed administration potentially enhances efficacy and reduces toxicity. Biomarker development is essential for identifying biomarkers that predict response to co-stimulatory agonists, guiding patient selection and optimizing treatment strategies. Biomarkers, such as ICOS expression, GITR density on Tregs, or OX40 expression on effector T cells, may help guide the use of these therapies in the clinic [46]. Nevertheless, as in the case of checkpoint inhibitors, resistance to co-stimulatory receptor agonists may emerge due to various factors, including T cell exhaustion, immune suppression in the TME, or upregulation of alternative immune checkpoints, therefore requiring innovative combination strategies and a deeper understanding of T cell biology.

Co-stimulatory receptor agonists represent a promising approach to enhancing T cell activation and improving outcomes in melanoma patients [47]. While early clinical trials have demonstrated encouraging results, further research is needed to optimize combination strategies, to reduce toxicities, and to identify predictive biomarkers. As the field of immunotherapy continues to evolve, co-stimulatory receptor agonists are likely to play a crucial role in next generation treatments in melanoma, particularly for patients who are resistant to current checkpoint blockade therapies.

However, it is important to mention that ICIs, including anti-CTLA-1 and PD-1, are fast-growing therapeutic approaches which display a spectrum of immune-related adverse effects (irAEs). A large spectrum of irAEs can affect different organs, cutaneous irAEs being reported in one out of three patients that received ICIs [48]. Patients treated with ipilimumab frequently display colitis [49], while pulmonary irAEs are marked as frequent in patients receiving anti PD-1 and anti-CTLA-4 antibodies [50]. Also, irAEs related to thyroid, hepatic, and cardiac tissues are observed after ICI therapies, along with rheumatological, neurological, and hematological irAEs [51].

**Table 2:** Alternative immune checkpoints inhibitors and co-stimulatory receptors agonists.

	Therapy	Mechanism of Action	Clinical Trial ID	Start Year	Model	Key Findings	Limitations	Ref.
Preclinical Study	Anti-VISTA	VISTA antagonistic mAb	N/A	2014	Animal Models: - C57BL/6 mice - OTII CD4 transgenic mice Tumor Models: - MB49, B16OVA, and B16BL6 tumor cells Genetic Model: - PTEN/BRAF melanoma induced by tamoxifen-activated recombinase	- Delayed tumor growth. - ↑ infiltration of CD8+ and CD4+ T cells into the TME. - ↑ activation of dendritic cells. - ↓ tumor-infiltrating MDSCs.	Limited to preclinical models; translation to human settings unproven.	[52]
	OX40 agonistic antibodies plus GSK2636771 (PI3Kβ inhibition)	OX40 agonism + PI3Kβ inhibition	N/A	2020	Cell lines: Human Mel2399, Mel2559, and autologous patients-derived TILs; Murine MC38/gp100 Animal models: transgenic murine melanoma models C57BL/6 mice and C57BL/6 albino mice (Braf-mutant, PTEN null)	- Potential to enhance T cell immunity and inhibit tumor growth via dual-targeted approach.	Limited to preclinical models; translation to human settings unproven.	[53]
	RMP1-30 (anti-PD-1 Ab), 8B.2C12 (anti-Tim-3 Ab)	PD-1 blockade + Tim-3 inhibition	N/A	2010	6–8-wk-old female BALB/c or C57BL/6 mice models	- Combination showing potential for enhanced immune checkpoint inhibition.	Incomplete clinical results.	[34]
	DTA-1 (agonist anti-GITR mAb)	GITR agonism	N/A	2010	Animal models: C57BL/6 Thy1.2+ and Thy1.1+ mice, Thy1.1+ pmel-1 T cell receptor transgenic mice	- Early indications of enhanced T cell activation and immune responses in melanoma.	Incomplete clinical data.	[54]
	9H10 Ab(anti-CTLA4), IVAX (ICOSL-expressing tumor vaccine)	CTLA-4 blockade, Immune modulation via ICOSL	N/A	2014	Animal model: 6-wk-old C57BL/6 and ICOS−/− mice	- Early preclinical evidence of enhanced immune activation and antitumor efficacy.	Uncertain translatability to human subjects.	[44]
Clinical Trials	CDX-1140 + Pembrolizumab	CD40 agonism + PD-1 blockade	NCT03329950	2017	Advanced/metastatic solid tumors	- Promising preliminary efficacy in solid tumors including ocular melanoma.	Phase I; safety still under evaluation.	[55]
	Epacadostat + Pembrolizumab	IDO-1 inhibition to counter tumor immune evasion	NCT02752074	2016	Advanced/metastatic melanoma	- Initial signals of synergy; later failed to meet endpoints.	High-profile failure in Phase III.	[56]
	SD-101 + Pembrolizumab	Immune activation via TLR9 agonism	NCT02521870	2015	Advanced/metastatic melanoma	- Enhanced intratumoral immune responses in early data.	Data limited to early-phase trials.	[57]
	T-VEC + Pembrolizumab	Oncolytic virus therapy + PD-1 blockade	NCT02263508	2014	Advanced/metastatic melanoma	Improved ORR and potential immune priming effects in early data.	OS benefit not conclusively shown.	[58]
	Sabatolimab + Spartalizumab	PD-1 blockade	NCT02608268	2015	Advanced/metastatic solid tumors	- Early evidence of synergistic immune modulation with potential for enhanced antitumor immunity.	Limited melanoma-specific data.	[59]
	9B12 (murine agonistic anti-human OX40 mAb)	OX40 agonism	NCT01644968	2003	Advanced/metastatic solid tumors	- Stimulated robust T cell activation and tumor regression in preclinical models.	Not humanized; limited clinical data.	[40]

## 3. Melanoma Vaccines

Not all melanomas exhibit a high tumor mutational burden (TMB), particularly subtypes such as acral, mucosal, and ocular melanomas. Additionally, the response rates to immune checkpoint inhibitors (ICIs) vary significantly based on the metastatic site, with lung metastases showing better outcomes compared to liver metastases, and even more so compared to adrenal and splenic metastases. Importantly, nearly 50% of melanomas remain incurable with current ICIs. This underscores the critical need for innovative approaches to address the challenges of low antigenicity and immunologically “cold” tumor microenvironments. Emerging strategies include melanoma vaccines, oncolytic viruses, bispecific T cell engagers, antibody–drug conjugates, and T cell therapies, all of which aim to overcome these barriers and enhance the efficacy of immunotherapy in melanoma treatment [60].

### 3.1. RNA Neoantigen Vaccines in Melanoma

RNA-based vaccines have gained attention due to their ability to induce robust immune responses. They involve synthesizing RNA sequences that encode melanoma-specific neoantigens, which are then administered to patients. Once inside the cells, the mRNA is translated into proteins that mimic the tumor neoantigens, leading to the activation of T cells that recognize and destroy melanoma cells. A groundbreaking study by Sahin et al. demonstrated the efficacy of RNA neoantigen vaccines in melanoma patients [61]. In this study, 13 patients with advanced melanoma received personalized RNA vaccines encoding up to 10 neoantigens identified through the genomic sequencing of their tumors. The vaccines induced strong T cell responses against the neoantigens, leading to complete or partial tumor regression in several patients. Remarkably, two patients who had a metastatic melanoma remained cancer-free after 23 months, showcasing the potential long-term efficiency of RNA vaccines in melanoma.

Another significant study by Ott et al. evaluated the safety and efficacy of personalized RNA neoantigen vaccines in patients with advanced melanoma. In this trial, six patients received vaccines targeting multiple neoantigens based on their individual tumor mutations [62]. The results were encouraging, with four patients showing no evidence of disease progression after treatment. Moreover, the vaccine induced polyclonal T cell responses, targeting multiple neoantigens, which enhances the likelihood of durable immune control over melanoma.

Neoantigen vaccines have also been tested in combination with immune checkpoint inhibitors, such as anti-PD-1 antibodies, further amplifying their efficacy. Studies have demonstrated the potential of combining neoantigen vaccines with immune checkpoint inhibitors (ICIs) to improve treatment outcomes for melanoma patients. For instance, the NeoVax vaccine has shown efficacy in generating sustained immune responses and long-term survival benefits in melanoma patients when combined with PD-1 inhibitors such as pembrolizumab. In a Phase Ib trial, the combination of the NEO-PV-01 neoantigen vaccine with pembrolizumab-enhanced neoantigen-specific CD4+ and CD8+ T cell responses and improved clinical outcomes in advanced melanoma patients [62]. The results were highly positive, with most patients demonstrating enhanced tumor regression. The combination therapy increased T cell infiltration into the TME, leading to prolonged disease-free survival in several patients. This study underscores the potential of combining RNA vaccines with other immunotherapies to improve the outcome in melanoma.

Neoantigen vaccines can be responsible for several side effects including fatigue, headache, nausea, diarrhea or pyrexia, which represent a normal reaction in this type of therapy. The patients that were included in trials with neoantigen vaccines were carefully monitored and the immune-mediated adverse events were low [63].

### 3.2. Dendritic Cell-Based RNA Vaccines

In addition to direct RNA vaccines, dendritic cell-based vaccines are another promising strategy. In this case, DCs are loaded with RNA-encoding melanoma neoantigens. Once reintroduced into the patient, DCs present neoantigens to T cells, triggering a targeted immune response. A notable study by Carreno et al. demonstrated positive outcomes with this approach. In this study, melanoma patients received dendritic cell vaccines loaded with neoantigen mRNA. The results showed that the vaccines were well-tolerated and effectively induced T cell response. T cells were able to recognize and destroy melanoma cells in vitro, and some patients experienced stable disease or partial responses. This study highlighted the potential of RNA-loaded DCs to elicit potential immune responses against melanoma [64]. While peptide- and antigen-focused vaccines like GVAX (GM-CSF-secreting vaccine) [65] or Melan-A/MART-1 and gp100 Peptides [66] are being explored, dendritic cell vaccines have not shown sufficient promise to remain a focus in melanoma immunotherapy.

### 3.3. Personalized RNA Vaccines: The Future of Melanoma Treatment

The success of personalized RNA vaccines lies in their ability to tailor treatment to the specific mutational landscape of each patient’s tumor. Unlike traditional vaccines targeting shared antigens, RNA vaccines can be designed to include a patient’s unique set of tumor neoantigens, making the immune response more precise and potent.

A recent phase I clinical trial used a viral vector to deliver neoantigen-encoded peptides, designed to optimize antigen presentation and elicit robust T cell responses. While NeoVax has been evaluated as a standalone therapy with occasional immune checkpoint inhibitor (ICI) use, NOUS-PEV is deliberately combined with pembrolizumab to assess synergy in advanced or metastatic melanoma. NeoVax demonstrated durable immune memory and epitope spreading in earlier-stage disease, whereas NOUS-PEV focuses on enhancing anti-tumor immunity in more aggressive settings, aiming to overcome resistance to ICIs. These differences underscore the expanding strategies in immunotherapy to tailor interventions according to the disease stage and immune context [67].

Additionally, personalized vaccines can be modified as the tumor evolves, allowing for a more dynamic and adaptive treatment approach, a key advantage in treating a cancer as heterogeneous as melanoma is. Despite the positive results, challenges remain in the development and widespread use of melanoma vaccines. One of the main hurdles is the identification of the most immunogenic neoantigens, as not all mutations generate strong immune responses. Furthermore, vaccine production can be time consuming and expensive, particularly for personalized therapies [68,69].

Nonetheless, advancements in bioinformatics, sequencing technologies, and RNA synthesis are making personalized vaccine development faster and more accessible. Future studies are expected on optimizing the selection of neoantigens and integrating vaccines with other therapies like immune checkpoint inhibitors, adoptive T cell therapy, and oncolytic viruses.

## 4. Oncolytic Viruses in Melanoma

### 4.1. Talimogene Laherparepvec (T-VEC)

The use of oncolytic viruses (OVs) as therapeutic agents in melanoma has gained significant momentum following the success of Talimogene laherparepvec (T-VEC). T-VEC is an oncolytic viral therapy derived from a genetically modified herpes simplex virus type-1 (HSV-1). It is one of the most significant breakthroughs in the treatment of melanoma, particularly for patients with unresectable metastatic disease. T-VEC works by selectively infecting and killing cancer cells while promoting a robust anti-tumor immune response [70]. T-VEC’s dual mechanism of action distinguishes it from other melanoma therapies. The virus is engineered to replicate selectively in tumor cells, sparing normal tissues. It also induces cell lysis, which releases tumor-derived antigens into the TME. Additionally, T-VEC is modified to express granulocyte-macrophage colony-stimulating factor (GM-CSF), which enhances local immune responses by recruiting DCs to process and present tumor antigens to cytotoxic T lymphocytes (CTLs). This leads to both a direct oncolytic effect and the initiation of systemic anti-tumor immunity, potentially targeting distant metastases [71]. T-VEC monotherapy showed promising results, with favorable efficacy and safety, displayed no dose-limiting toxicity and just common side effects as fever and chills, with most of the side effects considered as grade 1 and 2 [72,73].

One of the pivotal clinical trials that established T-VEC’s efficacy in melanoma is the OPTiM phase III trial. In this study, 436 patients with stage IIIB-IV melanoma were randomized to receive either intra-tumoral T-VEC injections or GM-CSF. The results demonstrated a durable response rate (DRR) of 16.3% in the T-VEC group compared to 2.1% in the GM-CSF group, indicating a significant improvement in long-term tumor control [74]. Additionally, overall survival (OS) was improved in patients with stage IIIB and IIIC disease who received T-VEC, with a median OS of 23.3 months compared to 18.9 months for those in the GM-CSF arm [75]. This trial provided the foundation for T-VEC’s FDA approval in 2015 for the treatment of unresectable, recurrent melanoma. Further analysis from the OPTiM trial highlighted the enhanced benefit of T-VEC in patients with less advanced melanoma (stage IIIB, IIIC, and IV-M1a). A subgroup analysis revealed that these patients experienced an improved response rate and overall survival when treated with T-VEC compared to patients with more advanced disease (stage IV-M1b/c). This finding suggests that T-VEC may be more effective in treating patients with injectable cutaneous, subcutaneous, or nodal lesions rather than visceral metastases, which are more challenging to control with intra-tumoral therapies [75].

### 4.2. Combination Therapy with Checkpoint Inhibitors

While T-VEC alone has shown significant benefits, its combination with immune checkpoint inhibitors has garnered considerable attention in recent studies. The combination of T-VEC with anti-PD-1 or anti-CTLA-4 antibodies can potentiate immune responses, as the local oncolysis induced by T-VEC primes the immune system, which may synergize with checkpoint blockade.

A phase Ib trial by Ribas et al. investigated the combination of T-VEC and PD-1 inhibitor pembrolizumab in patients with advanced melanoma. This therapeutical strategy demonstrated an objective response rate (ORR) of 62%, which is considerably higher than either agent alone. The combination was also well tolerated, with no unexpected toxicities [76]. These results led to the phase III, randomized, double-blind MASTERKEY-265/KEYNOTE-034 study, in patients with unresectable stage III-IVM1c melanoma who were naive to anti-PD1 therapy. The results of the MASTERKEY-265 trial indicated that combining talimogene laherparepvec (T-VEC) with pembrolizumab did not provide a significant improvement in progression-free survival (PFS) or overall survival (OS) compared to pembrolizumab alone in patients with advanced melanoma. Despite some numerical improvements in secondary endpoints, such as objective response rates and durable response rates, these were not statistically significant. The study’s findings highlighted the need for additional combination strategies to improve efficacy, especially for patients resistant to anti-PD-1 treatment [77].

Preclinical studies have been instrumental in understanding T-VEC’s mechanisms and optimizing its use in melanoma therapy. One study by Liu et al. used murine melanoma models to demonstrate that T-VEC treatment led to an increase in tumor-infiltrating CD8+ T cells and to a reduction in immunosuppressive Tregs within the TME. This shift toward a more pro-inflammatory and immune-reactive microenvironment supports the clinical observation of T-VEC’s ability to trigger systemic immune responses and control metastatic disease [78].

In addition, several preclinical models have examined the role of T-VEC in promoting DCs’ maturation and in enhancing the presentation of melanoma-associated antigens to the immune system. Kaufman et al. highlighted the ability of T-VEC to induce robust DCs’ recruitment and activation, leading to a stronger anti-tumor immune response [78,79]. The immune activation could be further enhanced when T-VEC is combined with immune checkpoint blockade or adoptive cell therapies, suggesting a versatile role for T-VEC in the evolving landscape of melanoma treatment.

Despite the promising clinical outcomes, there are several limitations in the use of T-VEC for melanoma treatment. One limitation is the reliance on injectable, accessible tumors for intra-tumoral administration, which makes it less effective in patients with visceral metastases or non-injectable disease. Additionally, the response to T-VEC tends to be limited to patients with relatively low tumor burden, as those with extensive or rapidly progressing disease may not derive the same benefits. Moreover, there is ongoing research to optimize the delivery of T-VEC and other oncolytic viruses for metastatic melanoma. Studies are investigating the use of alternative administration routes, including intravenous delivery or engineered virus strains with enhanced tumor-targeting capabilities [80].

### 4.3. Oncolytic Viruses in Melanoma: Beyond T-VEC

While T-VEC is the first FDA-approved oncolytic virus for melanoma, other oncolytic viruses, such as vaccinia virus, coxsackievirus, reovirus, Newcastle disease virus (NDV), and adenovirus are being explored in preclinical and clinical trials for their potential to selectively kill melanoma cells and stimulate anti-tumor immunity.

The vaccinia virus is one of the most studied oncolytic viruses due to its large genome, which allows the insertion of therapeutic genes, and its natural tropism for cancer cells. Modified vaccinia viruses have shown great promise in melanoma treatment. A notable example is the JX-594 strain, which is engineered to express GM-CSF. In a phase I-II clinical trial, JX-594 demonstrated safety and efficacy in patients with advanced melanoma. The virus was well-tolerated, and patients showed tumor necrosis and immune cell infiltration into tumor tissues, indicating an immune response [81]. Moreover, it was demonstrated that JX-594 is capable of selectively infecting cancer tissues upon intravenous infusion, in a dose-dependent manner, while healthy tissues were not affected. Nevertheless, it also showed anti-tumor activity for over 4 weeks of disease control in patients affected by melanoma [82].

Coxsackievirus A21 (CVA21) is a naturally occurring oncolytic enterovirus that selectively targets and lyses melanoma cells due to its affinity for intercellular adhesion molecule-1 (ICAM-1) and decay-accelerating factor (DAF), which are normally overexpressed on melanoma cells. A phase II clinical trial, CALM, evaluated CVA21 in patients with stage IIIc-IV melanoma and showed a 28% objective response rate (ORR), with a durable complete response in several cases [83]. Furthermore, CVA21 was found to be safe, with mild flu-like symptoms being the most common side effect. The virus also demonstrated the ability to prime systemic anti-tumor immunity, resulting in the clearance of non-injected lesions, which represents a hallmark of a strong immune response. Preclinical studies have suggested that CVA21 can further improve outcomes when combined with immune checkpoint inhibitors. One study showed that the combination of CVA21 and anti-PD-1 therapy in a murine melanoma model significantly improved survival rates compared to either treatment alone [84].

Reovirus is a double-stranded RNA virus that naturally targets and replicates in cancer cells with an activated Ras pathway, a common feature in melanoma. Reolysin^®^, a proprietary formulation of reovirus, has been tested in both preclinical and clinical studies for melanoma. In a phase I/II clinical trial, Reolysin was combined with carboplatin and paclitaxel in patients with metastatic melanoma. The study reported promising results, with a 31% ORR, and several patients achieving partial or complete responses [85]. Additionally, the virus was shown to induce tumor necrosis and promote immune cell infiltration, similar to other oncolytic viruses (OVs). Reovirus also demonstrated significant efficacy in preclinical melanoma models, where it was found to induce immunogenic cell death and stimulate anti-tumor immunity [86]. Studies have suggested that reovirus works best when used in combination with other treatments, such as chemotherapy or checkpoint inhibitors, to enhance its therapeutic effects.

Newcastle disease virus (NDV) is a paramyxovirus that selectively infects and kills tumor cells while sparing healthy cells. NDV has been studied for its oncolytic potential in melanoma and other cancers. A clinical study involving intra-tumoral NDV injections in melanoma patients demonstrated safety and modest efficacy, with a few patients experiencing tumor regression [87]. Although the clinical response rates were lower compared to T-VEC, NDV showed the ability to induce strong immune responses, which can be harnessed for combination therapies. In preclinical studies, NDV was found to activate DCs and promote the maturation of tumor-specific T cells, making it a potent immune-stimulating agent. NDV’s ability to modulate the immune system makes it an attractive candidate for combination with immune checkpoint inhibitors or adoptive T cell therapies [80].

Adenoviruses are another class of oncolytic viruses being explored for melanoma treatment due to their modifiability and their ability to infect a wide range of cell types. ONCOS-102 is a genetically engineered adenovirus that expresses GM-CSF and it was designed to selectively target tumor cells while enhancing anti-tumor immune responses. A phase I clinical trial of ONCOS-102 in patients with advanced melanoma reported a 33% ORR, with some patients achieving long-lasting responses [88]. The virus was also found to increase tumor-specific T cell responses, suggesting its potential to induce durable anti-tumor immunity. Preclinical studies have shown that adenoviruses can be engineered to express additional immune-stimulatory molecules, further enhancing their therapeutic potential [89]. When used in combination with immune checkpoint inhibitors, adenoviruses have been shown to significantly improve survival in melanoma models, highlighting their potential for combination therapies.

### 4.4. Combination Therapies with Oncolytic Viruses

The success of T-VEC has opened the door to exploring other OVs in combination with immune checkpoint inhibitors, targeted therapies, or even adoptive T cell therapies. One of the key advantages of OVs is their ability to convert “cold” tumors, which lack immune infiltration, into “hot” tumors, which are more responsive to immunotherapy. Studies have shown that OVs can enhance the efficacy of immune checkpoint inhibitors by increasing the infiltration of T cells into tumors and promoting the release of tumor antigens, thus amplifying the immune response. In melanoma, combination therapies involving OVs and immune checkpoint inhibitors have shown great promise in both preclinical and clinical settings. As mentioned above, the combination of T-VEC with pembrolizumab demonstrated superior efficacy compared to either agent alone, providing a template for future studies with different Ovs [80]. The use of OVs in melanoma therapy clearly is a rapidly evolving field. As largely demonstrated, these viruses not only directly kill tumor cells but also stimulate robust anti-tumor immune responses, making them ideal candidates for combination therapies [90]. Their ability to enhance immune infiltration into tumors and promote systemic anti-tumor immunity makes them a valuable addition to the growing arsenal of immunotherapies for melanoma.

## 5. Bispecific T Cell Engagers (BiTEs) and Antibody–Drug Conjugates (ADCs)

In recent years, bispecific T cell engagers (BiTEs) and antibody–drug conjugates (ADCs) have emerged as promising therapeutic options for melanoma and other cancers. These innovative treatments harness the body’s immune system or targeted cytotoxic mechanisms, making them crucial in the treatment of tumors resistant to traditional therapies.

Patients that receive BiTEs or antibody–drug conjugates can develop complications including CRS, ICANS, liver injury, and secondary infections due to immunosuppressive therapies used to reduce toxicity [91,92,93].

BiTEs are engineered molecules that recruit cytotoxic T cells to the TME. These molecules simultaneously bind a tumor-associated antigen (TAA) to melanoma cells and CD3 to T cells, facilitating proper proximity that leads to T cell activation and subsequent tumor cell lysis. Their ability to directly engage the immune system offers a promising mechanism to eradicate cancer cells, even those which are otherwise evasive to immune surveillance. A standout example of BiTEs in melanoma is the one targeting melanoma-associated antigen A1 (MAGE-A1). MAGE-A1-specific BiTEs displayed high cytotoxicity against melanoma cells expressing this antigen. Indeed, these BiTEs could effectively redirect T cells to kill melanoma cells both in vitro and in animal models, offering a new approach to target melanoma-associated antigens not addressed previously by conventional therapies [94]. The potency of MAGE-A1-specific BiTEs was influenced by the epitope’s distance from the target cell membrane and the antigen size, with closer proximity and smaller antigen size enhancing T cell-mediated lysis. Therefore, optimizing these factors can improve the clinical effectiveness of BiTE therapies targeting melanoma [95]. This approach opens avenues for melanoma patients who express antigens beyond the most well-known ones like PD-L1 or CTLA-4. Tebentafusp (also known as IMCgp100) is a promising bispecific T cell engager (BiTE) that has shown significant clinical efficacy in uveal melanoma, particularly in patients with metastatic disease. Tebentafusp works by targeting the gp100 antigen, which is overexpressed in melanoma cells, and redirecting T cells to kill these cells. This treatment represents a novel approach to uveal melanoma, a subtype of melanoma with limited therapeutic options, and has demonstrated improved progression-free survival (PFS) and overall survival (OS). In clinical trials, success in uveal melanoma has been highlighted; its discussion has mainly been reserved for the conclusions of many studies, possibly due to its relatively specific application to this melanoma subtype [96].

An exciting avenue of research is the ongoing exploration of BiTEs targeting other melanoma-associated antigens, such as the PRAME (preferentially expressed antigen in melanoma) antigen. Unlike gp100, which is more commonly expressed in cutaneous melanoma, PRAME has shown promise as a target in cutaneous melanoma and other melanoma subtypes. PRAME is a target for BiTE therapy because it is frequently overexpressed in various cancers, including melanoma, but its expression is limited in normal tissues, which may reduce potential off-target effects and increase the therapeutic index of treatments. Current studies involving PRAME-specific BiTEs in cutaneous melanoma are building on the success seen with gp100-based therapies, and they may offer new options for patients who do not respond to conventional treatments like immune checkpoint inhibitors. These studies are essential for expanding the scope of BiTE therapy beyond uveal melanoma, as they hold promise for improving outcomes in the more common cutaneous melanoma as well. Given the importance of expanding BiTE therapy’s applicability, these ongoing studies with PRAME as an antigen deserve more attention and citation. As we gain a better understanding of how targeting PRAME can enhance T cell-mediated tumor destruction, it may become a pivotal approach in treating cutaneous melanoma and possibly other cancers with high PRAME expression [97].

One of the key advantages of BiTEs in melanoma therapy is their potential to overcome immunosuppressive mechanisms that melanoma cells use to evade immune destruction. As T cell redirection does not rely on the patient’s pre-existing immunity, BiTEs can generate robust antitumor responses even in cases where immune checkpoints or T cell exhaustion have limited other immunotherapies [98,99]. Alternatively, a significant limitation in the clinical application of BiTEs (bispecific T cell engagers) is their HLA (human leukocyte antigen) haplotype restriction, which can limit their efficacy across diverse patient populations. HLA molecules play a crucial role in presenting antigens to T cells, and BiTEs typically rely on the recognition of these antigens by T cell receptors (TCRs) in an HLA-dependent manner. As a result, BiTE therapies are often restricted to patients who express specific HLA alleles that can present the target antigen effectively to the immune system [100,101].

This HLA haplotype restriction becomes a substantial hurdle, particularly in genetically heterogeneous populations. Different individuals possess varying HLA genotypes, and the binding affinity of BiTE antibodies to their target HLA class I or II molecules can vary significantly across these genotypes. Consequently, BiTE-based therapies may show varying levels of effectiveness depending on a patient’s HLA profile, with patients who lack the appropriate allele unable to respond to the treatment, thus limiting the universality of this approach in cancer therapy [102,103,104].

Several studies have highlighted this limitation in immune-based therapies. For example, research on T cell engagers targeting melanoma-associated antigens, such as MAGE-A3 or NY-ESO-1, has shown that the presence of specific HLA alleles, such as HLA-A2, is crucial for the therapeutic effectiveness of these treatments and reduces the potential for widespread application of BiTE therapies, especially in populations with diverse or underrepresented HLA haplotypes. Moreover, this HLA restriction contrasts with the more universal targeting mechanisms seen in other cancer therapies, such as checkpoint inhibitors, which do not depend on a specific HLA type for efficacy. Therefore, researchers have been exploring ways to overcome this limitation, including the development of BiTEs that can recognize non-HLA-restricted target structures or use allogeneic T cell therapies that bypass the need for specific HLA compatibility. The HLA haplo action remains a critical factor that needs to be addressed in future research to maximize the therapeutic potential of BiTEs and similar immune-modulating treatments, ensuring that they can be broadly effective across different patient populations.

ADCs are another breakthrough class of therapeutics. These agents consist of an antibody directed toward a specific TAA, linked to a cytotoxic drug. Upon binding to the antigen in melanoma cells, the conjugated drug is internalized within the tumor cells, leading to their destruction. ADCs combine the specificity of targeted therapy with the potency of chemotherapy, limiting off-target effects while increasing efficacy.

In melanoma, ADCs targeting TAAs like gpNMB and c-Met have shown promising results. Glembatumumab vedotin (CDX-011), targeting glycoprotein NMB (gpNMB), a protein overexpressed in advanced melanoma, has been tested in clinical trials. The study by Yardley et al. demonstrated that this ADC yielded significant tumor shrinkage in a subset of patients with metastatic melanoma expressing high levels of gpNMB. This result was especially promising for patients who had progressed on prior immunotherapies, highlighting the potential of ADCs in treating refractory melanoma cases [105,106].

### Combining BiTEs and ADCs with Other Therapies

Both BiTEs and ADCs may benefit from combination strategies with other melanoma treatments, such as immune checkpoint inhibitors or targeted therapies, like BRAF and MEK inhibitors. For instance, combination of BiTE with anti-PD-1 antibodies enhanced antitumor activity in a melanoma model. This synergy was particularly impressive since it addresses both the immune suppression and heterogeneity commonly seen in melanoma tumors, potentially broadening the patient population that can benefit from BiTEs [107].

Similarly, ADCs can be subjected to combinations with immunotherapies. In the case of Glembatumumab vedotin, when combined with immune checkpoint inhibitors, an enhanced therapeutic efficacy has been illustrated in preclinical models [45]. By integrating ADCs into multi-modal treatment regimens, melanoma therapy can be further refined to improve both response rates and durability.

Although BiTEs and ADCs represent exciting advances in melanoma treatment, certain challenges are to be taken into consideration. One major obstacle is the potential for off-target effects and the development of resistance mechanisms. For the use of BiTEs, T cell exhaustion and tumor immune escape remain potential issues, especially in highly immunosuppressive TME. Strategies to mitigate these conditions, such as combining BiTEs with checkpoint inhibitors or other immune-modulating agents, are actively being explored. For the use of ADCs, a significant hurdle is achieving optimal drug delivery to the tumor without affecting normal tissues. Ongoing research focuses on improving the linker chemistry and drug payloads to increase the selectivity and potency of these agents. Moreover, understanding the mechanisms of resistance to ADCs, such as the downregulation of target antigens or drug efflux, is critical to optimizing their therapeutic potential. BiTEs and ADCs are emerging as powerful tools in the fight against melanoma, particularly for patients who have developed resistance to traditional therapies [96,108,109].

As research continues to advance, combining BiTEs and ADCs with other therapies could further improve outcomes and provide new hope for patients with advanced melanoma.

## 6. TCR-T and TIL Therapies in Melanoma

T cell receptor-engineered T cells (TCR-T) and tumor-infiltrating lymphocytes (TIL) therapies have shown great promise in harnessing and enhancing the body’s immune response to target melanoma. These advanced treatments focus on recognizing tumor-specific antigens and reinvigorating T cells to attack melanoma cells. TCR-T therapy involves genetically modifying a patient’s T cells to express T cell receptors (TCRs) that target tumor-associated antigens (TAAs) or tumor-specific neoantigens. Once infused back into the patient, these engineered T cells seek out and destroy melanoma cells that express the targeted antigen.

A pioneering study by Morgan et al. demonstrated the potential of TCR-T in melanoma by using TCR-modified T cells specific to MART-1, a melanoma-associated antigen. Two patients with advanced melanoma experienced significant tumor regression, marking an important step in the development of TCR-T for cancer treatment [110]. More recently, Robbins et al. showed that 11 out of 20 patients with metastatic melanoma, treated with TCR-T cells targeting NY-ESO-1, achieved partial or complete tumor regression. This was particularly encouraging for patients who had previously failed immunotherapies, highlighting the potential of TCR-T in resistant melanoma cases [111].

TIL therapy involves isolating tumor-reactive lymphocytes from a patient’s tumor and expanding them ex vivo before reinfusion. Since these lymphocytes already recognize cancer cells, they can mount a robust immune attack when reintroduced in larger numbers.

The first TIL therapy study by Rosenberg et al. (2011) emphasizes that despite prior treatments with high-dose IL-2, 5 out of 11 heavily pretreated melanoma patients achieved either complete or partial responses (CR + PR) with adoptive T cell transfer. This suggests that T cell therapy can still offer therapeutic benefits in patients with advanced melanoma who have undergone multiple lines of prior therapy, including IL-2. One key aspect to comment on is the resilience of T cell-based therapies even in the face of immune suppression or prior exhaustion induced by IL-2, which has been known for its profound effects on the immune system. The success observed may also be indicative of the persistence and adaptability of the immune cells engineered for the therapy. However, given the small sample size, caution is needed in generalizing these results across larger patient populations [112]. Recent randomized trial involved 101 patients with metastatic melanoma. The trial tested different lymphodepletion regimens and examined the effect of TIL infusion. The study found durable complete responses (CR) in 24% of patients, and a significant portion of patients (45–62%) exhibited objective responses. This study confirmed that the combination of TIL infusion with lymphodepleting chemotherapy could have promising results, although the best treatment protocols still require optimization [113]. Long-term follow-up revealed durable disease-free survival in many patients, extending beyond five years in certain cases. Additionally, a subsequent phase II clinical trial by Goff et al. reported a 56% ORR in patients with metastatic melanoma, many of whom had progressed after immune checkpoint inhibitor therapy [113]. The study by Rohaan et al. (2022) employed a rigorous and thoughtful design to evaluate the comparative effectiveness of tumor-infiltrating lymphocyte (TIL) therapy and ipilimumab in patients with advanced melanoma. Patients eligible for the trial had metastatic disease and had not responded to prior immune checkpoint inhibitor therapy. To enable the TIL process, a sample of each patient’s tumor was surgically resected. From this tissue, TILs were isolated and expanded in the laboratory under carefully controlled conditions, utilizing interleukin-2 (IL-2) and additional feeder cells to cultivate a robust population of these immune cells. This method aimed to maximize the therapeutic potential of TILs before their reinfusion into the patient. Before receiving the TIL infusion, patients underwent a preparatory lymphodepleting regimen using cyclophosphamide and fludarabine. This conditioning step was designed to create a favorable immune environment for the infused TILs to persist and function effectively. After the TIL infusion, patients received high-dose IL-2 to further stimulate the activity and survival of the transferred cells. The control arm of the study administered ipilimumab, a well-established CTLA-4 inhibitor, at the standard therapeutic dosage and schedule. The study’s endpoints focused on progression-free survival as the primary outcome, along with secondary measures such as overall response rate, overall survival, safety, and quality of life. TIL therapy yielded significantly higher objective response rates, emphasizing its ability to leverage patient-specific tumor-derived immune cells for precise and effective tumor targeting. Furthermore, progression-free survival was notably extended in the TIL-treated group, marking a substantial advance in controlling disease progression. Despite requiring intensive preparatory regimens, including lymphodepletion and high-dose IL-2, the safety profile of TIL therapy was manageable and consistent with known immunotherapy toxicities [114]. These results solidify the effectiveness of TIL therapy on refractory melanoma.

### Enhancing TCR-T and TIL Therapies with Combination Approaches

Combining TCR-T and TIL therapies with other immunotherapies, such as immune checkpoint inhibitors, has shown potential in improving treatment outcomes by overcoming T cell exhaustion and enhancing antitumor activity. Combination of TIL therapy with anti-PD-1 therapy resulted in higher response rates compared to either treatment alone, offering a synergistic approach to reinvigorating TILs in the TME [115].

Similarly, a trial by Johnson et al. found that combining TCR-T targeting NY-ESO-1 with pembrolizumab led to enhanced T cell activation and prolonged tumor regression in metastatic melanoma. This combination represents a promising strategy for improving outcomes in melanoma treatment [116].

Despite the positive results of TCR-T and TIL therapies, it is important to state the current limitations. Identifying the most effective tumor antigens for targeting is critical, as not all patients express the same antigens. Additionally, the immunosuppressive TME can limit T cell activity, leading to therapeutic resistance. Ongoing research aims to optimize TCR affinity, improve T cell expansion methods, and identify new targets to increase the efficacy of TCR-T therapy. Advances in cell culture techniques and the use of cytokines like IL-2 are improving TIL therapy, enhancing the expansion and functionality of tumor-reactive lymphocytes [117].

The use of TCR-T and TILs should take into consideration the risk of adverse effects such as neurological symptoms, neurotoxicity and CRS in different grades, as antigens may not be tumor specific and increase the risk of adverse events [118].

In summary, TCR-T and TIL therapies have demonstrated substantial efficacy in melanoma treatment, particularly in patients with advanced or treatment-resistant cases. Clinical trials by Morgan et al. [110], Robbins et al. [111] and Rosenberg et al. [112] have highlighted the capacity of these therapies to induce durable tumor regression, even in patients who have failed other treatments. Combining these adoptive cell therapies with immune checkpoint inhibitors has further improved their effectiveness, offering new hope for patients with metastatic melanoma.

## 7. CAR-T Cell Therapy in Melanoma

CAR-T therapy involves genetically engineering T cells to express a synthetic receptor (CAR) that can recognize specific tumor-associated antigens independently of MHC presentation. Once reintroduced into the patient, these modified T cells bind to the tumor antigen and activate an immune response aimed at destroying tumor cells. While CAR-T has shown success in treating blood cancers, targeting solid tumors like melanoma presents challenges due to antigen heterogeneity, the immunosuppressive TME and T cell exhaustion.

One of the earliest studies on CAR-T therapy in melanoma was conducted by Schaft et al. [119], where human T cells were engineered to express a CAR targeting the melanoma-associated antigen gp100. These modified T cells successfully targeted and lysed melanoma cells in vitro and in mouse models, establishing a foundation for future clinical studies.

Early-phase clinical trials of CAR-T therapy in melanoma have yielded mixed but promising results. A promising trial by Pule et al. involved CAR-T cells targeting GD2 in patients with relapsed or refractory melanoma. Despite a small sample size, notable reductions in tumor size were observed in two patients, while others maintained a prolonged stable disease [120]. This outcome underscores the potential of targeting antigens like GD2 in melanoma.

A significant challenge in CAR-T therapy for melanoma is tumor antigen heterogeneity. To address this, researchers are exploring the use of CAR-T cells engineered to target multiple antigens simultaneously, thereby enhancing their effectiveness in eliminating melanoma cells [121,122]. Additionally, Kloss et al. investigated bispecific CAR-T cells targeting HER2 and IL13Rα2 in melanoma models [123]. These bispecific CAR-T cells showed improved tumor control and longer survival in mouse models compared to monospecific CAR-T cells, suggesting they may help tackle melanoma’s antigenic heterogeneity.

The immunosuppressive TME poses another obstacle for CAR-T cell therapy in melanoma, inhibiting T cell function and reducing CAR-T persistence. Several studies have explored methods to enhance CAR-T survival and activity in this hostile environment. Moon et al. engineered CAR-T cells to secrete IL-12, a cytokine that promotes T cell activation and can counteract immunosuppression. In melanoma mouse models, these IL-12-secreting CAR-T cells exhibited significantly enhanced antitumor activity compared to non-secreting CAR-T cells, demonstrating that modifying CAR-T cells to release pro-inflammatory cytokines can improve efficacy [124]. Moreover, preclinical trials by Newick et al. investigated the use of checkpoint blockade to enhance CAR-T cell persistence and function in melanoma. Combination of CAR-T therapy with PD-1 blockade improved T cell persistence and greater antitumor effects were observed in mouse models, a strategy now being tested in clinical trials [125]. Another study is examining CAR-T cells engineered to target B7-H3, an immune checkpoint molecule overexpressed in melanoma. Early data indicate that B7-H3-targeting CAR-T cells may help overcome challenges related to T cell exhaustion and improve therapeutic outcomes [126].

The use of CAR T cells in different malignancies was debated by multiple groups, and the main impediments that were reported include immune-effector-cell-associated neurotoxicity syndrome (ICANS), tumor lysis syndrome, on-target/off-tumor toxicity, and cytokine release syndromes (CRS). Thus, patients receiving CAR T cells need to be carefully monitored [127].

The encouraging results from these studies suggest that CAR-T therapy may play a significant role in future melanoma treatments, especially when combined with strategies to target multiple antigens and overcome the immunosuppressive TME. Innovations such as bispecific CARs, induced cytokine secretion, and immune checkpoint inhibition present exciting avenues for improving CAR-T therapy efficacy in melanoma.

## 8. CAR/TCR-T or Bispecific T Cell Engagers in Melanoma? Can One Improve upon the Efficacy of Natural Cancer-Cognate Lymphocytes?

Among these new therapeutic approaches that are explored, Chimeric Antigen Receptor T cells (CAR-T), T Cell Receptor-engineered T cells (TCR-T), and bispecific T cell engagers (BiTEs) stand out as the most promising options. Each of these immunotherapies holds the potential to revolutionize melanoma treatment by redirecting the patient’s immune system toward cancer cells [128,129,130]. Yet, a fundamental question arises: can these sophisticated approaches improve upon the efficacy of natural cancer-cognate lymphocytes?

### 8.1. CAR-T Therapy in Melanoma: Promise and Challenges

Applying CAR-T cell therapy to solid tumors like melanoma has proven to be more difficult due to the tumor microenvironment and antigen heterogeneity.

Early preclinical studies have given hints on the efficacy of CAR-T cells in melanoma. For example, Chinnasamy et al. designed CAR-T cells to target VEGFR-2, an angiogenesis-related protein highly expressed in melanoma [131]. The CAR-T cells showed strong antitumor activity in preclinical models, causing regression of melanoma tumors. Despite these promising results, translating these effects into the clinic has been challenging. Moreover, tumor antigen escape, limited persistence of CAR-T cells, and immunosuppressive factors in the TME remain significant hurdles. Another limitation of CAR-T in melanoma can be antigen selection [132]. Melanoma expresses various tumor-associated antigens, but many are shared with normal tissues, increasing the risk of off-target toxicities.

### 8.2. TCR-T Cells: Targeting Melanoma with Precision

TCR-T therapy differs from CAR-T in that T cells are engineered to express receptors recognizing peptides presented by MHC molecules, allowing for more precise targeting of intracellular tumor-associated antigens. This makes TCR-T therapy particularly appealing for melanoma, which expresses numerous neoantigens due to its high mutational burden [133]. A stating example is the previously mentioned study by Robbins et al., in which patients with metastatic melanoma were treated with TCR-T cells targeting NY-ESO-1, a cancer-testis antigen [111]. The trial reported objective responses in 55% of patients, with some experiencing partial or complete tumor regression (Anti-NY ESO-1 TCR PBL trial, protocol number 08-C-0121N). Importantly, TCR-T therapy showed efficacy even in patients who had progressed after immune checkpoint inhibitors, suggesting its potential as a second-line therapy.

The ability of TCR-T therapy to target intracellular antigens that are not accessible to CAR-T cells offers an advantage in treating melanoma. Moreover, TCR-T cells can recognize a broader range of epitopes, making them more adaptable to the heterogeneous antigen landscape in melanoma. However, TCR-T therapy implicates its limitations, particularly concerning MHC restriction, which limits the applicability to patients with specific HLA types [134]. Additionally, like CAR-T, TCR-T cells face challenges related to the immunosuppressive TME.

### 8.3. Bispecific T Cell Engagers (BiTEs): Redirecting T Cells to Kill Melanoma

Bispecific T cell engagers (BiTEs) act as a bridge between T cells and tumor cells. BiTEs consist of two linked antibodies: one that binds to a tumor-specific antigen and another that binds to CD3 on T cells, activating them to kill tumor cells. Unlike CAR-T and TCR-T cells, BiTEs do not require genetic engineering of T cells and can redirect both endogenous and adoptively transferred T cells [135].

One of the most extensively studied BiTEs in melanoma targets the gp100 antigen. A preclinical study by Huehls et al. showed that BiTEs targeting gp100 could effectively redirect T cells to melanoma cells and induce significant tumor cell death. The flexibility of BiTEs in using the patient’s own T cells to target melanoma offers an advantage, particularly in settings where T cells are still functional but have been exhausted by the TME [136].

Another advantage of BiTEs is their ability to act immediately upon infusion, without the need for extensive T cell expansion ex vivo. However, BiTEs typically have short half-lives, requiring continuous infusion to maintain their therapeutic efficacy. The success of BiTEs in melanoma has yet to match their efficacy in hematological malignancies, but ongoing trials are exploring different antigen targets and BiTE designs to enhance their effectiveness in melanoma [109].

### 8.4. The Best Cancer Killer: Cancer-Cognate Lymphocytes?

One of the primary strengths of cancer-cognate lymphocytes, such as tumor-infiltrating lymphocytes (TILs), is their natural ability to recognize tumor antigens and persist within the TME. The efficiency of TILs therapy and their durable response has been demonstrated in metastatic melanoma patients, with some experiencing complete remission [112]. The key advantage of TILs lies in their ability to recognize a broad array of tumor antigens, which may include both known TAAs and patient-specific neoantigens.

However, the use of TILs presents limitations, particularly in heavily pretreated patients whose T cells may be functionally exhausted. In these cases, therapies like CAR/TCR-T and BiTEs offer alternative approaches to reinvigorating the immune response [137,138].

### 8.5. CAR/TCR-T or BiTEs—Which Is the Best Strategy?

**Figure 3:**
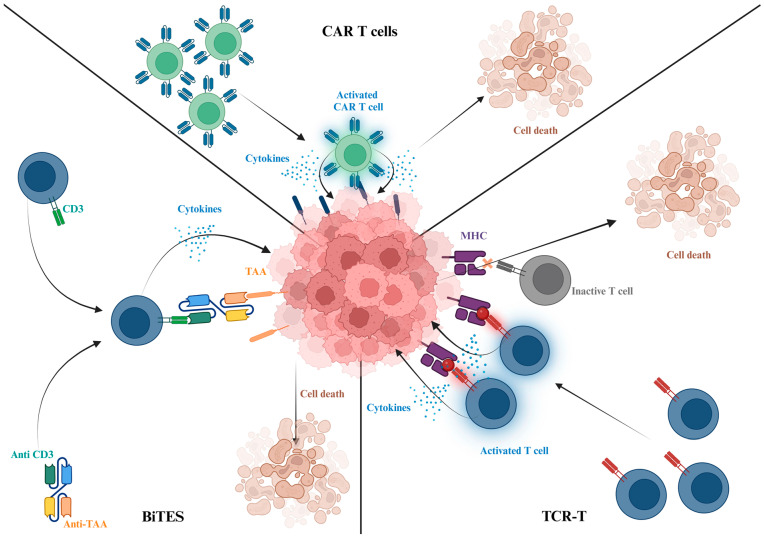
CAR T vs. BiTEs vs. TCR-T therapies in melanoma. CAR T targeting surface antigens on tumor cells. TCR-T binding to the tumor antigen presented by MHC. BiTEs engage in the interaction between T cells and tumor cells.

CAR-T, TCR-T, and BiTE therapies (Figure 3) each offer unique advantages in the treatment of melanoma, but none have yet surpassed the natural cancer-killing capabilities of cancer-cognate lymphocytes like TILs. TCR-T cells have shown the most promise due to their ability to target intracellular antigens and neoantigens, while CAR-T cells are hampered by antigen escape and the challenges of targeting melanoma’s heterogeneous antigens. BiTEs offer a simpler, off-the-shelf approach but may lack the persistence and efficacy needed for long-term control of melanoma [139].

Ultimately, the most effective strategy may involve combining these approaches to create a multi-faceted attack on melanoma. Trials combining CAR-T or TCR-T cells with immune checkpoint inhibitors or using BiTEs to enhance the activity of adoptively transferred T cells are already underway and may show promise in overcoming the limitations of each therapy alone [140,141,142,143]. The future of melanoma treatment likely lies in harnessing the strengths of both engineered therapies and cancer-cognate lymphocytes, proving that there may indeed be no better cancer killer than a naturally occurring T cell—enhanced by the best that biotechnology has to offer.

## 9. Conclusions

Despite the transformative advances brought by anti-PD-1/L1 and anti-CTLA-4 therapies, achieving a universal breakthrough in cancer immunotherapy remains challenging. The multifaceted nature of cancer immunity, combined with the diversity of tumor microenvironments (TMEs) and cancer types, complicates the development of broadly effective treatments. These complexities highlight the necessity of personalized treatment strategies tailored to individual tumor profiles and immune landscapes to maximize patient outcomes.

Melanoma cancer cells leverage a range of immune checkpoints, such as LAG-3, TIM-3, and TIGIT, in addition to PD-1 and CTLA-4, to evade immune detection and destruction. These molecules suppress T cell activity and foster an immunosuppressive environment that facilitates tumor survival. Emerging therapies targeting these checkpoints have demonstrated potential in preclinical models, suggesting synergistic benefits when combined with existing checkpoint inhibitors. However, the use of agonist receptors in combination therapies is often limited by their specific toxicities. This underscores the need to carefully balance efficacy and safety in designing combination regimens.

The mechanisms of resistance to immune checkpoint inhibitors (ICIs) are varied and multifactorial. Tumor-related factors include tumor mutational burden (TMB), neoantigen presentation, and vascularization. T cell-related mechanisms, such as exhaustion or immune depletion, also play a critical role. The tumor microenvironment (TME) further compounds resistance through the presence of myeloid-derived suppressor cells (MDSCs), tumor-associated macrophages (TAMs), and vascular endothelial growth factor (VEGF). Patient-specific factors, including comorbidities like diabetes, chronic inflammation, or the need for immunosuppressive drugs, add another layer of complexity. Overcoming these diverse resistance mechanisms requires tailored strategies targeting each underlying cause.

A critical resistance mechanism within the TME involves MDSCs, which suppress T cell activation and promote tumor growth through the secretion of immunosuppressive factors. Targeting MDSCs or their associated signaling pathways could offer new therapeutic avenues, particularly for patients who fail to respond to current immunotherapies. Similarly, agents modulating innate immunity, such as co-stimulatory receptor agonists like CD28 and OX40, represent promising strategies, especially when paired with checkpoint inhibitors to amplify anti-tumor immunity.

Therapeutic innovation alone, however, is insufficient. The unequal accessibility and availability of advanced immunotherapies worldwide significantly restricts their impact. High costs, regulatory barriers, and infrastructure limitations prevent many patients from benefiting from cutting-edge treatments. Addressing these disparities is critical, as improved global access could drastically increase the number of curable patients. For instance, tebentafusp’s approval for HLA-A*02:01-positive uveal melanoma represents a milestone but is limited by HLA haplotype specificity and geographic disparities in access. Broadening availability and tailoring these treatments to diverse patient populations could significantly enhance cure rates.

Ultimately, the path forward lies in integrating advanced biomarker discovery and multi-omics approaches to identify patient-specific targets, enabling the design of truly personalized therapies. By addressing immunosuppressive mechanisms like MDSCs, refining combination regimens to mitigate toxicity, and ensuring equitable access globally, cancer immunotherapy can move closer to realizing its transformative potential.

## References

[1] Klempner S.J., Fabrizio D., Bane S., Reinhart M., Peoples T., Ali S.M., Sokol E.S., Frampton G., Schrock A.B., Anhorn R. (2020). Tumor Mutational Burden as a Predictive Biomarker for Response to Immune Checkpoint Inhibitors: A Review of Current Evidence. Oncologist.

[2] Dousset L., Poizeau F., Robert C., Mansard S., Mortier L., Caumont C., Routier E., Dupuy A., Rouanet J., Battistella M. (2021). Positive Association Between Location of Melanoma, Ultraviolet Signature, Tumor Mutational Burden, and Response to Anti-PD-1 Therapy. JCO Precis. Oncol..

[3] Eroglu Z., Zaretsky J.M., Hu-Lieskovan S., Kim D.W., Algazi A., Johnson D.B., Liniker E., Ben K., Munhoz R., Rapisuwon S. (2018). High response rate to PD-1 blockade in desmoplastic melanomas. Nature.

[4] Daya-Grosjean L., Dumaz N., Sarasin A. (1995). The specificity of p53 mutation spectra in sunlight induced human cancers. J. Photochem. Photobiol. B.

[5] Pfeifer G.P., Besaratinia A. (2012). UV wavelength-dependent DNA damage and human non-melanoma and melanoma skin cancer. Photochem. Photobiol. Sci..

[6] Laughery M.F., Wilson H.E., Sewell A., Stevison S., Wyrick J.J. (2024). The Surprising Diversity of UV-Induced Mutations. Adv. Genet..

[7] Trucco L.D., Mundra P.A., Hogan K., Garcia-Martinez P., Viros A., Mandal A.K., Macagno N., Gaudy-Marqueste C., Allan D., Baenke F. (2019). Ultraviolet radiation-induced DNA damage is prognostic for outcome in melanoma. Nat. Med..

[8] Schumacher T.N., Schreiber R.D. (2015). Neoantigens in cancer immunotherapy. Science.

[9] Hugo W., Zaretsky J.M., Sun L., Song C., Moreno B.H., Hu-Lieskovan S., Berent-Maoz B., Pang J., Chmielowski B., Cherry G. (2016). Genomic and Transcriptomic Features of Response to Anti-PD-1 Therapy in Metastatic Melanoma. Cell.

[10] Bowman R.L., Hennessey R.C., Weiss T.J., Tallman D.A., Crawford E.R., Murphy B.M., Webb A., Zhang S., La Perle K.M., Burd C.J. (2021). UVB mutagenesis differs in Nras- and Braf-mutant mouse models of melanoma. Life Sci. Alliance.

[11] Reis B., Attig J., Dziadek S., Graefe N., Heller A., Rieder N., Gomes B. (2024). Tumor beta2-microglobulin and HLA-A expression is increased by immunotherapy and can predict response to CIT in association with other biomarkers. Front. Immunol..

[12] Falcone I., Conciatori F., Bazzichetto C., Ferretti G., Cognetti F., Ciuffreda L., Milella M. (2020). Tumor Microenvironment: Implications in Melanoma Resistance to Targeted Therapy and Immunotherapy. Cancers.

[13] Van Allen E.M., Miao D., Schilling B., Shukla S.A., Blank C., Zimmer L., Sucker A., Hillen U., Foppen M.H.G., Goldinger S.M. (2015). Genomic correlates of response to CTLA-4 blockade in metastatic melanoma. Science.

[14] Tucci M., Passarelli A., Mannavola F., Felici C., Stucci L.S., Cives M., Silvestris F. (2019). Immune System Evasion as Hallmark of Melanoma Progression: The Role of Dendritic Cells. Front. Oncol..

[15] Taube J.M., Klein A., Brahmer J.R., Xu H., Pan X., Kim J.H., Chen L., Pardoll D.M., Topalian S.L., Anders R.A. (2014). Association of PD-1, PD-1 ligands, and other features of the tumor immune microenvironment with response to anti-PD-1 therapy. Clin. Cancer Res..

[16] Campbell K.M., Amouzgar M., Pfeiffer S.M., Howes T.R., Medina E., Travers M., Steiner G., Weber J.S., Wolchok J.D., Larkin J. (2023). Prior anti-CTLA-4 therapy impacts molecular characteristics associated with anti-PD-1 response in advanced melanoma. Cancer Cell.

[17] Colucci M., D’Alonzo V., Santangelo F., Miracco C., Valente M., Maio M., Di Giacomo A.M. (2022). Successful Targeting of CTLA-4 in a Melanoma Clinical Case: A Long-Term “One Stop Therapeutic Shop”. Onco Targets Ther..

[18] Kaunitz G.J., Cottrell T.R., Lilo M., Muthappan V., Esandrio J., Berry S., Xu H., Ogurtsova A., Anders R.A., Fischer A.H. (2017). Melanoma subtypes demonstrate distinct PD-L1 expression profiles. Lab. Investig..

[19] Gajewski T.F., Schreiber H., Fu Y.X. (2013). Innate and adaptive immune cells in the tumor microenvironment. Nat. Immunol..

[20] Fridman W.H., Pages F., Sautes-Fridman C., Galon J. (2012). The immune contexture in human tumours: Impact on clinical outcome. Nat. Rev. Cancer.

[21] Chen D.S., Mellman I. (2017). Elements of cancer immunity and the cancer-immune set point. Nature.

[22] Raskova M., Lacina L., Kejik Z., Venhauerova A., Skalickova M., Kolar M., Jakubek M., Rosel D., Smetana K., Brabek J. (2022). The Role of IL-6 in Cancer Cell Invasiveness and Metastasis-Overview and Therapeutic Opportunities. Cells.

[23] Grivennikov S.I., Greten F.R., Karin M. (2010). Immunity, inflammation, and cancer. Cell.

[24] Feig C., Jones J.O., Kraman M., Wells R.J., Deonarine A., Chan D.S., Connell C.M., Roberts E.W., Zhao Q., Caballero O.L. (2013). Targeting CXCL12 from FAP-expressing carcinoma-associated fibroblasts synergizes with anti-PD-L1 immunotherapy in pancreatic cancer. Proc. Natl. Acad. Sci. USA.

[25] Rausch M.P., Hastings K.T., Ward W.H., Farma J.M. (2017). Immune Checkpoint Inhibitors in the Treatment of Melanoma: From Basic Science to Clinical Application. Cutaneous Melanoma: Etiology and Therapy, Brisbane (AU).

[26] Chow M.T., Ozga A.J., Servis R.L., Frederick D.T., Lo J.A., Fisher D.E., Freeman G.J., Boland G.M., Luster A.D. (2019). Intratumoral Activity of the CXCR3 Chemokine System Is Required for the Efficacy of Anti-PD-1 Therapy. Immunity.

[27] Fenton S.E., Sosman J.A., Chandra S. (2019). Resistance mechanisms in melanoma to immuneoncologic therapy with checkpoint inhibitors. Cancer Drug Resist..

[28] Hodi F.S., O’Day S.J., McDermott D.F., Weber R.W., Sosman J.A., Haanen J.B., Gonzalez R., Robert C., Schadendorf D., Hassel J.C. (2010). Improved survival with ipilimumab in patients with metastatic melanoma. N. Engl. J. Med..

[29] Robert C., Long G.V., Brady B., Dutriaux C., Maio M., Mortier L., Hassel J.C., Rutkowski P., McNeil C., Kalinka-Warzocha E. (2015). Nivolumab in previously untreated melanoma without BRAF mutation. N. Engl. J. Med..

[30] Robert C., Schachter J., Long G.V., Arance A., Grob J.J., Mortier L., Daud A., Carlino M.S., McNeil C., Lotem M. (2015). Pembrolizumab versus Ipilimumab in Advanced Melanoma. N. Engl. J. Med..

[31] Wolchok J.D., Chiarion-Sileni V., Gonzalez R., Rutkowski P., Grob J.J., Cowey C.L., Lao C.D., Wagstaff J., Schadendorf D., Ferrucci P.F. (2017). Overall Survival with Combined Nivolumab and Ipilimumab in Advanced Melanoma. N. Engl. J. Med..

[32] Tawbi H.A., Schadendorf D., Lipson E.J., Ascierto P.A., Matamala L., Castillo Gutierrez E., Rutkowski P., Gogas H.J., Lao C.D., De Menezes J.J. (2022). Relatlimab and Nivolumab versus Nivolumab in Untreated Advanced Melanoma. N. Engl. J. Med..

[33] Ascierto P.A., Lipson E.J., Dummer R., Larkin J., Long G.V., Sanborn R.E., Chiarion-Sileni V., Dreno B., Dalle S., Schadendorf D. (2023). Nivolumab and Relatlimab in Patients With Advanced Melanoma That Had Progressed on Anti-Programmed Death-1/Programmed Death Ligand 1 Therapy: Results From the Phase I/IIa RELATIVITY-020 Trial. J. Clin. Oncol..

[34] Sakuishi K., Apetoh L., Sullivan J.M., Blazar B.R., Kuchroo V.K., Anderson A.C. (2010). Targeting Tim-3 and PD-1 pathways to reverse T cell exhaustion and restore anti-tumor immunity. J. Exp. Med..

[35] Lin C.C., Curigliano G., Santoro A., Kim D.W., Tai D., Hodi F.S., Wilgenhof S., Doi T., Sabatos-Peyton C., Szpakowski S. (2024). Sabatolimab in combination with spartalizumab in patients with non-small cell lung cancer or melanoma who received prior treatment with anti-PD-1/PD-L1 therapy: A phase 2 multicentre study. BMJ Open.

[36] Chauvin J.M., Pagliano O., Fourcade J., Sun Z., Wang H., Sander C., Kirkwood J.M., Chen T.H., Maurer M., Korman A.J. (2015). TIGIT and PD-1 impair tumor antigen-specific CD8(+) T cells in melanoma patients. J. Clin. Investig..

[37] Tang W., Chen J., Ji T., Cong X. (2023). TIGIT, a novel immune checkpoint therapy for melanoma. Cell Death Dis..

[38] Vesely M.D., Kidacki M., Gaule P., Gupta S., Chan N.N.N., Han X., Yeung J.T., Chen L. (2024). Immune Inhibitory Molecule PD-1 Homolog (VISTA) Colocalizes with CD11b Myeloid Cells in Melanoma and Is Associated with Poor Outcomes. J. Investig. Dermatol..

[39] Yum J.I., Hong Y.K. (2021). Terminating Cancer by Blocking VISTA as a Novel Immunotherapy: Hasta la vista, baby. Front. Oncol..

[40] Curti B.D., Kovacsovics-Bankowski M., Morris N., Walker E., Chisholm L., Floyd K., Walker J., Gonzalez I., Meeuwsen T., Fox B.A. (2013). OX40 is a potent immune-stimulating target in late-stage cancer patients. Cancer Res..

[41] Chester C., Sanmamed M.F., Wang J., Melero I. (2018). Immunotherapy targeting 4-1BB: Mechanistic rationale, clinical results, and future strategies. Blood.

[42] Hall M.S., Mullinax J.E., Cox C.A., Hall A.M., Beatty M.S., Blauvelt J., Innamarato P., Nagle L., Branthoover H., Wiener D. (2022). Combination Nivolumab, CD137 Agonism, and Adoptive Cell Therapy with Tumor-Infiltrating Lymphocytes for Patients with Metastatic Melanoma. Clin. Cancer Res..

[43] Schaer D.A., Murphy J.T., Wolchok J.D. (2012). Modulation of GITR for cancer immunotherapy. Curr. Opin. Immunol..

[44] Fan X., Quezada S.A., Sepulveda M.A., Sharma P., Allison J.P. (2014). Engagement of the ICOS pathway markedly enhances efficacy of CTLA-4 blockade in cancer immunotherapy. J. Exp. Med..

[45] Wu R.C., Luke J.J. (2024). Uncovering the Potential of CD40 Agonism to Enhance Immune Checkpoint Blockade. Clin. Cancer Res..

[46] van der Sluis T.C., Beyrend G., van der Gracht E.T.I., Abdelaal T., Jochems S.P., Belderbos R.A., Wesselink T.H., van Duikeren S., van Haften F.J., Redeker A. (2023). OX40 agonism enhances PD-L1 checkpoint blockade by shifting the cytotoxic T cell differentiation spectrum. Cell Rep. Med..

[47] Jeong S., Park S.H. (2020). Co-Stimulatory Receptors in Cancers and Their Implications for Cancer Immunotherapy. Immune Netw..

[48] Villadolid J., Amin A. (2015). Immune checkpoint inhibitors in clinical practice: Update on management of immune-related toxicities. Transl. Lung Cancer Res..

[49] Dougan M. (2017). Checkpoint Blockade Toxicity and Immune Homeostasis in the Gastrointestinal Tract. Front. Immunol..

[50] Chuzi S., Tavora F., Cruz M., Costa R., Chae Y.K., Carneiro B.A., Giles F.J. (2017). Clinical features, diagnostic challenges, and management strategies in checkpoint inhibitor-related pneumonitis. Cancer Manag. Res..

[51] Martins F., Sofiya L., Sykiotis G.P., Lamine F., Maillard M., Fraga M., Shabafrouz K., Ribi C., Cairoli A., Guex-Crosier Y. (2019). Adverse effects of immune-checkpoint inhibitors: Epidemiology, management and surveillance. Nat. Rev. Clin. Oncol..

[52] Le Mercier I., Chen W., Lines J.L., Day M., Li J., Sergent P., Noelle R.J., Wang L. (2014). VISTA Regulates the Development of Protective Antitumor Immunity. Cancer Res..

[53] Peng W., Williams L.J., Xu C., Melendez B., McKenzie J.A., Chen Y., Jackson H.L., Voo K.S., Mbofung R.M., Leahey S.E. (2019). Anti-OX40 Antibody Directly Enhances The Function of Tumor-Reactive CD8(+) T Cells and Synergizes with PI3Kbeta Inhibition in PTEN Loss Melanoma. Clin. Cancer Res..

[54] Cohen A.D., Schaer D.A., Liu C., Li Y., Hirschhorn-Cymmerman D., Kim S.C., Diab A., Rizzuto G., Duan F., Perales M.A. (2010). Agonist anti-GITR monoclonal antibody induces melanoma tumor immunity in mice by altering regulatory T cell stability and intra-tumor accumulation. PLoS ONE.

[55] Sanborn R., Gabrail N., Carneiro B., O’Hara M., Bordoni R., Gordon M., Khalil D., Hauke R., Taglienti C., Rogalski M. (2022). 596 Results from a phase 1 study of CDX-1140, a fully human anti-CD40 agonist monoclonal antibody (mAb), in combination with pembrolizumab. J. Immuno Ther. Cancer.

[56] Long G.V., Dummer R., Hamid O., Gajewski T.F., Caglevic C., Dalle S., Arance A., Carlino M.S., Grob J.J., Kim T.M. (2019). Epacadostat plus pembrolizumab versus placebo plus pembrolizumab in patients with unresectable or metastatic melanoma (ECHO-301/KEYNOTE-252): A phase 3, randomised, double-blind study. Lancet Oncol..

[57] Cohen E.E.W., Nabell L., Wong D.J.L., Day T.A., Daniels G.A., Milhem M.M., Deva S., Jameson M.B., Guntinas-Lichius O., Almubarak M. (2019). Phase 1b/2, open label, multicenter study of intratumoral SD-101 in combination with pembrolizumab in anti-PD-1 treatment naïve patients with recurrent or metastatic head and neck squamous cell carcinoma (HNSCC). J. Clin. Oncol..

[58] Long G., Dummer R., Johnson D., Michielin O., Martin-Algarra S., Treichel S., Chan E., Diede S., Ribas A. (2020). 429 Long-term analysis of MASTERKEY-265 phase 1b trial of talimogene laherparepvec (T-VEC) plus pembrolizumab in patients with unresectable stage IIIB-IVM1c melanoma. J. Immuno Ther. Cancer.

[59] Curigliano G., Gelderblom H., Mach N., Doi T., Tai D., Forde P.M., Sarantopoulos J., Bedard P.L., Lin C.C., Hodi F.S. (2021). Phase I/Ib Clinical Trial of Sabatolimab, an Anti-TIM-3 Antibody, Alone and in Combination with Spartalizumab, an Anti-PD-1 Antibody, in Advanced Solid Tumors. Clin. Cancer Res..

[60] Alexandrov L.B., Nik-Zainal S., Wedge D.C., Aparicio S.A., Behjati S., Biankin A.V., Bignell G.R., Bolli N., Borg A., Borresen-Dale A.L. (2013). Signatures of mutational processes in human cancer. Nature.

[61] Sahin U., Derhovanessian E., Miller M., Kloke B.P., Simon P., Lower M., Bukur V., Tadmor A.D., Luxemburger U., Schrors B. (2017). Personalized RNA mutanome vaccines mobilize poly-specific therapeutic immunity against cancer. Nature.

[62] Ott P.A., Hu Z., Keskin D.B., Shukla S.A., Sun J., Bozym D.J., Zhang W., Luoma A., Giobbie-Hurder A., Peter L. (2017). An immunogenic personal neoantigen vaccine for patients with melanoma. Nature.

[63] Weber J.S., Carlino M.S., Khattak A., Meniawy T., Ansstas G., Taylor M.H., Kim K.B., McKean M., Long G.V., Sullivan R.J. (2024). Individualised neoantigen therapy mRNA-4157 (V940) plus pembrolizumab versus pembrolizumab monotherapy in resected melanoma (KEYNOTE-942): A randomised, phase 2b study. Lancet.

[64] Carreno B.M., Magrini V., Becker-Hapak M., Kaabinejadian S., Hundal J., Petti A.A., Ly A., Lie W.R., Hildebrand W.H., Mardis E.R. (2015). Cancer immunotherapy. A dendritic cell vaccine increases the breadth and diversity of melanoma neoantigen-specific T cells. Science.

[65] Dillman R. (2020). An update on GM-CSF and its potential role in melanoma management. Melanoma Manag..

[66] Di Pucchio T., Pilla L., Capone I., Ferrantini M., Montefiore E., Urbani F., Patuzzo R., Pennacchioli E., Santinami M., Cova A. (2006). Immunization of stage IV melanoma patients with Melan-A/MART-1 and gp100 peptides plus IFN-alpha results in the activation of specific CD8(+) T cells and monocyte/dendritic cell precursors. Cancer Res..

[67] D’Alise A.M., Leoni G., Cotugno G., Siani L., Vitale R., Ruzza V., Garzia I., Antonucci L., Micarelli E., Venafra V. (2024). Phase I Trial of Viral Vector-Based Personalized Vaccination Elicits Robust Neoantigen-Specific Antitumor T-Cell Responses. Clin. Cancer Res..

[68] Bafaloukos D., Gazouli I., Koutserimpas C., Samonis G. (2023). Evolution and Progress of mRNA Vaccines in the Treatment of Melanoma: Future Prospects. Vaccines.

[69] Yang R., Cui J. (2024). Advances and applications of RNA vaccines in tumor treatment. Molecular Cancer.

[70] Bayan C.Y., Lopez A.T., Gartrell R.D., Komatsubara K.M., Bogardus M., Rao N., Chen C., Hart T.D., Enzler T., Rizk E.M. (2018). The Role of Oncolytic Viruses in the Treatment of Melanoma. Curr. Oncol. Rep..

[71] Rehman H., Silk A.W., Kane M.P., Kaufman H.L. (2016). Into the clinic: Talimogene laherparepvec (T-VEC), a first-in-class intratumoral oncolytic viral therapy. J. Immunother. Cancer.

[72] Yamazaki N., Koga H., Kojima T., Tsutsumida A., Namikawa K., Yi M., Mera K., Pickett-Gies C. (2018). Early safety from a phase I, multicenter, open-label, dose de-escalation study of talimogene laherparepvec (T-VEC) in Japanese patients (pts) with unresectable stage IIIB-IV melanoma (MEL). Ann. Oncol..

[73] Zhang T., Jou T.H., Hsin J., Wang Z., Huang K., Ye J., Yin H., Xing Y. (2023). Talimogene Laherparepvec (T-VEC): A Review of the Recent Advances in Cancer Therapy. J. Clin. Med..

[74] Andtbacka R.H., Kaufman H.L., Collichio F., Amatruda T., Senzer N., Chesney J., Delman K.A., Spitler L.E., Puzanov I., Agarwala S.S. (2015). Talimogene Laherparepvec Improves Durable Response Rate in Patients With Advanced Melanoma. J. Clin. Oncol..

[75] Andtbacka R.H.I., Collichio F., Harrington K.J., Middleton M.R., Downey G., Ohrling K., Kaufman H.L. (2019). Final analyses of OPTiM: A randomized phase III trial of talimogene laherparepvec versus granulocyte-macrophage colony-stimulating factor in unresectable stage III-IV melanoma. J. Immunother. Cancer.

[76] Ribas A., Dummer R., Puzanov I., VanderWalde A., Andtbacka R.H.I., Michielin O., Olszanski A.J., Malvehy J., Cebon J., Fernandez E. (2017). Oncolytic Virotherapy Promotes Intratumoral T Cell Infiltration and Improves Anti-PD-1 Immunotherapy. Cell.

[77] Ribas A., Chesney J., Long G.V., Kirkwood J.M., Dummer R., Puzanov I., Hoeller C., Gajewski T.F., Gutzmer R., Rutkowski P. (2021). 1037O MASTERKEY-265: A phase III, randomized, placebo (Pbo)-controlled study of talimogene laherparepvec (T) plus pembrolizumab (P) for unresectable stage IIIB–IVM1c melanoma (MEL). Ann. Oncol..

[78] Liu B.L., Robinson M., Han Z.Q., Branston R.H., English C., Reay P., McGrath Y., Thomas S.K., Thornton M., Bullock P. (2003). ICP34.5 deleted herpes simplex virus with enhanced oncolytic, immune stimulating, and anti-tumour properties. Gene Ther..

[79] Kohlhapp F.J., Zloza A., Kaufman H.L. (1998). Talimogene Laherparepvec (T-VEC) as Cancer Immunotherapy. Drugs Today.

[80] Kaufman H.L., Kohlhapp F.J., Zloza A. (2015). Oncolytic viruses: A new class of immunotherapy drugs. Nat. Rev. Drug Discov..

[81] Park B.-H., Hwang T.-H., Kim S.-G., Rhee B.-G., Ahn Y.-J., Kwon H.-C., Oh S.-Y., Han S.-Y., Speth K., Crompton A.M. (2007). A phase I-II clinical trial with JX-594, a targeted and GM-CSF-armed oncolytic poxvirus, by intratumoral injection in patients with liver tumors. Mol. Cancer Ther..

[82] Breitbach C.J., Burke J., Jonker D., Stephenson J., Haas A.R., Chow L.Q., Nieva J., Hwang T.H., Moon A., Patt R. (2011). Intravenous delivery of a multi-mechanistic cancer-targeted oncolytic poxvirus in humans. Nature.

[83] Andtbacka R.H.I., Curti B.D., Kaufman H., Daniels G.A., Nemunaitis J.J., Spitler L.E., Hallmeyer S., Lutzky J., Schultz S., Whitman E.D. (2014). CALM study: A phase II study of an intratumorally delivered oncolytic immunotherapeutic agent, coxsackievirus A21, in patients with stage IIIc and stage IV malignant melanoma. J. Clin. Oncol..

[84] Curti B.D., Richards J.M., Hallmeyer S., Faries M.B., Andtbacka R.H.I., Daniels G.A., Grose M., Shafren D. (2017). Activity of a novel immunotherapy combination of intralesional Coxsackievirus A21 and systemic ipilimumab in advanced melanoma patients previously treated with anti-PD1 blockade therapy. J. Clin. Oncol..

[85] Mahalingam D., Fountzilas C., Moseley J., Noronha N., Tran H., Chakrabarty R., Selvaggi G., Coffey M., Thompson B., Sarantopoulos J. (2017). A phase II study of REOLYSIN((R)) (pelareorep) in combination with carboplatin and paclitaxel for patients with advanced malignant melanoma. Cancer Chemother. Pharmacol..

[86] Errington F., White C.L., Twigger K.R., Rose A., Scott K., Steele L., Ilett L.J., Prestwich R., Pandha H.S., Coffey M. (2008). Inflammatory tumour cell killing by oncolytic reovirus for the treatment of melanoma. Gene Ther..

[87] Zamarin D., Vigil A., Kelly K., Garcia-Sastre A., Fong Y. (2009). Genetically engineered Newcastle disease virus for malignant melanoma therapy. Gene Ther..

[88] Ranki T., Pesonen S., Hemminki A., Partanen K., Kairemo K., Alanko T., Lundin J., Linder N., Turkki R., Ristimaki A. (2016). Phase I study with ONCOS-102 for the treatment of solid tumors—An evaluation of clinical response and exploratory analyses of immune markers. J. Immunother. Cancer.

[89] Tseha S.T. (2022). Role of Adenoviruses in Cancer Therapy. Front. Oncol..

[90] Conry R.M., Westbrook B., McKee S., Norwood T.G. (2018). Talimogene laherparepvec: First in class oncolytic virotherapy. Hum. Vaccin Immunother..

[91] Salvaris R., Ong J., Gregory G.P. (2021). Bispecific Antibodies: A Review of Development, Clinical Efficacy and Toxicity in B-Cell Lymphomas. J. Pers. Med..

[92] Cosenza M., Sacchi S., Pozzi S. (2021). Cytokine Release Syndrome Associated with T-Cell-Based Therapies for Hematological Malignancies: Pathophysiology, Clinical Presentation, and Treatment. Int. J. Mol. Sci..

[93] Hansel T.T., Kropshofer H., Singer T., Mitchell J.A., George A.J. (2010). The safety and side effects of monoclonal antibodies. Nat. Rev. Drug Discov..

[94] Shivarov V., Blazhev G. (2021). Bringing Together the Power of T Cell Receptor Mimic and Bispecific Antibodies for Cancer Immunotherapy: Still a Long Way to Go. Monoclon. Antibodies Immunodiagn. Immunother..

[95] Bacac M., Klein C., Umana P. (2016). CEA TCB: A novel head-to-tail 2:1 T cell bispecific antibody for treatment of CEA-positive solid tumors. Oncoimmunology.

[96] Jin S., Sun Y., Liang X., Gu X., Ning J., Xu Y., Chen S., Pan L. (2022). Emerging new therapeutic antibody derivatives for cancer treatment. Signal Transduct. Target. Ther..

[97] Reschke R., Enk A.H., Hassel J.C. (2024). T Cell-Engaging Bispecific Antibodies Targeting gp100 and PRAME: Expanding Application from Uveal Melanoma to Cutaneous Melanoma. Pharmaceutics.

[98] Zieger N., Nicholls A., Wulf J., Hänel G., Kazerani Pasikhani M., Buecklein V., Brauchle B., Marcinek A., Nixdorf D., Rohrbacher L. (2020). Treatment-Free Intervals Mitigate T-Cell Exhaustion Induced By Continuous CD19xCD3-BiTE® Construct Stimulation in Vitro. Blood.

[99] Goebeler M.-E., Bargou R.C. (2020). T cell-engaging therapies—BiTEs and beyond. Nat. Rev. Clin. Oncol..

[100] de Miguel M., Calvo E. (2021). T cell engagers in solid tumors kick the door down. Cancer Cell.

[101] van de Donk N., Zweegman S. (2023). T-cell-engaging bispecific antibodies in cancer. Lancet.

[102] Holland C.J., Crean R.M., Pentier J.M., de Wet B., Lloyd A., Srikannathasan V., Lissin N., Lloyd K.A., Blicher T.H., Conroy P.J. (2020). Specificity of bispecific T cell receptors and antibodies targeting peptide-HLA. J. Clin. Investig..

[103] Pagliuca S., Gurnari C., Rubio M.T., Visconte V., Lenz T.L. (2022). Individual HLA heterogeneity and its implications for cellular immune evasion in cancer and beyond. Front. Immunol..

[104] Høydahl L.S., Berntzen G., Løset G.Å. (2024). Engineering T-cell receptor–like antibodies for biologics and cell therapy. Curr. Opin. Biotechnol..

[105] Yardley D.A., Weaver R., Melisko M.E., Saleh M.N., Arena F.P., Forero A., Cigler T., Stopeck A., Citrin D., Oliff I. (2015). EMERGE: A Randomized Phase II Study of the Antibody-Drug Conjugate Glembatumumab Vedotin in Advanced Glycoprotein NMB-Expressing Breast Cancer. J. Clin. Oncol..

[106] Ott P.A., Hamid O., Pavlick A.C., Kluger H., Kim K.B., Boasberg P.D., Simantov R., Crowley E., Green J.A., Hawthorne T. (2014). Phase I/II study of the antibody-drug conjugate glembatumumab vedotin in patients with advanced melanoma. J. Clin. Oncol..

[107] Fernandez M.F., Choi J., Sosman J. (2023). New Approaches to Targeted Therapy in Melanoma. Cancers.

[108] Sau S., Alsaab H.O., Kashaw S.K., Tatiparti K., Iyer A.K. (2017). Advances in antibody-drug conjugates: A new era of targeted cancer therapy. Drug Discov. Today.

[109] Tang J., Gong Y., Ma X. (2022). Bispecific Antibodies Progression in Malignant Melanoma. Front. Pharmacol..

[110] Morgan R.A., Dudley M.E., Wunderlich J.R., Hughes M.S., Yang J.C., Sherry R.M., Royal R.E., Topalian S.L., Kammula U.S., Restifo N.P. (2006). Cancer regression in patients after transfer of genetically engineered lymphocytes. Science.

[111] Robbins P.F., Morgan R.A., Feldman S.A., Yang J.C., Sherry R.M., Dudley M.E., Wunderlich J.R., Nahvi A.V., Helman L.J., Mackall C.L. (2011). Tumor regression in patients with metastatic synovial cell sarcoma and melanoma using genetically engineered lymphocytes reactive with NY-ESO-1. J. Clin. Oncol..

[112] Rosenberg S.A., Yang J.C., Sherry R.M., Kammula U.S., Hughes M.S., Phan G.Q., Citrin D.E., Restifo N.P., Robbins P.F., Wunderlich J.R. (2011). Durable complete responses in heavily pretreated patients with metastatic melanoma using T-cell transfer immunotherapy. Clin. Cancer Res..

[113] Goff S.L., Dudley M.E., Citrin D.E., Somerville R.P., Wunderlich J.R., Danforth D.N., Zlott D.A., Yang J.C., Sherry R.M., Kammula U.S. (2016). Randomized, Prospective Evaluation Comparing Intensity of Lymphodepletion Before Adoptive Transfer of Tumor-Infiltrating Lymphocytes for Patients With Metastatic Melanoma. J. Clin. Oncol..

[114] Rohaan M.W., Borch T.H., van den Berg J.H., Met O., Kessels R., Geukes Foppen M.H., Stoltenborg Granhoj J., Nuijen B., Nijenhuis C., Jedema I. (2022). Tumor-Infiltrating Lymphocyte Therapy or Ipilimumab in Advanced Melanoma. N. Engl. J. Med..

[115] van den Berg J.H., Heemskerk B., van Rooij N., Gomez-Eerland R., Michels S., van Zon M., de Boer R., Bakker N.A.M., Jorritsma-Smit A., van Buuren M.M. (2020). Tumor infiltrating lymphocytes (TIL) therapy in metastatic melanoma: Boosting of neoantigen-specific T cell reactivity and long-term follow-up. J. Immunother. Cancer.

[116] Johnson L.A., Morgan R.A., Dudley M.E., Cassard L., Yang J.C., Hughes M.S., Kammula U.S., Royal R.E., Sherry R.M., Wunderlich J.R. (2009). Gene therapy with human and mouse T-cell receptors mediates cancer regression and targets normal tissues expressing cognate antigen. Blood.

[117] Jiang T., Zhou C., Ren S. (2016). Role of IL-2 in cancer immunotherapy. Oncoimmunology.

[118] Liu Y., Yan X., Zhang F., Zhang X., Tang F., Han Z., Li Y. (2021). TCR-T Immunotherapy: The Challenges and Solutions. Front. Oncol..

[119] Schaft N., Willemsen R.A., de Vries J., Lankiewicz B., Essers B.W., Gratama J.W., Figdor C.G., Bolhuis R.L., Debets R., Adema G.J. (2003). Peptide fine specificity of anti-glycoprotein 100 CTL is preserved following transfer of engineered TCR alpha beta genes into primary human T lymphocytes. J. Immunol..

[120] Pule M.A., Savoldo B., Myers G.D., Rossig C., Russell H.V., Dotti G., Huls M.H., Liu E., Gee A.P., Mei Z. (2008). Virus-specific T cells engineered to coexpress tumor-specific receptors: Persistence and antitumor activity in individuals with neuroblastoma. Nat. Med..

[121] Daei Sorkhabi A., Mohamed Khosroshahi L., Sarkesh A., Mardi A., Aghebati-Maleki A., Aghebati-Maleki L., Baradaran B. (2023). The current landscape of CAR T-cell therapy for solid tumors: Mechanisms, research progress, challenges, and counterstrategies. Front. Immunol..

[122] Peng L., Sferruzza G., Yang L., Zhou L., Chen S. (2024). CAR-T and CAR-NK as cellular cancer immunotherapy for solid tumors. Cell. Mol. Immunol..

[123] Kloss C.C., Condomines M., Cartellieri M., Bachmann M., Sadelain M. (2013). Combinatorial antigen recognition with balanced signaling promotes selective tumor eradication by engineered T cells. Nat. Biotechnol..

[124] Moon E.K., Wang L.C., Dolfi D.V., Wilson C.B., Ranganathan R., Sun J., Kapoor V., Scholler J., Pure E., Milone M.C. (2014). Multifactorial T-cell hypofunction that is reversible can limit the efficacy of chimeric antigen receptor-transduced human T cells in solid tumors. Clin. Cancer Res..

[125] Newick K., Moon E., Albelda S.M. (2016). Chimeric antigen receptor T-cell therapy for solid tumors. Mol. Ther. Oncolytics.

[126] Zhang Z., Jiang C., Liu Z., Yang M., Tang X., Wang Y., Zheng M., Huang J., Zhong K., Zhao S. (2020). B7-H3-Targeted CAR-T Cells Exhibit Potent Antitumor Effects on Hematologic and Solid Tumors. Mol. Ther. Oncolytics.

[127] Zhang Y., Qin D., Shou A.C., Liu Y., Wang Y., Zhou L. (2023). Exploring CAR-T Cell Therapy Side Effects: Mechanisms and Management Strategies. J. Clin. Med..

[128] Alsajjan R., Mason W.P. (2023). Bispecific T-Cell Engagers and Chimeric Antigen Receptor T-Cell Therapies in Glioblastoma: An Update. Curr. Oncol..

[129] Tsimberidou A.-M., Van Morris K., Vo H.H., Eck S., Lin Y.-F., Rivas J.M., Andersson B.S. (2021). T-cell receptor-based therapy: An innovative therapeutic approach for solid tumors. J. Hematol. Oncol..

[130] Jilani S., Saco J.D., Mugarza E., Pujol-Morcillo A., Chokry J., Ng C., Abril-Rodriguez G., Berger-Manerio D., Pant A., Hu J. (2024). CAR-T cell therapy targeting surface expression of TYRP1 to treat cutaneous and rare melanoma subtypes. Nat. Commun..

[131] Chinnasamy D., Yu Z., Theoret M.R., Zhao Y., Shrimali R.K., Morgan R.A., Feldman S.A., Restifo N.P., Rosenberg S.A. (2010). Gene therapy using genetically modified lymphocytes targeting VEGFR-2 inhibits the growth of vascularized syngenic tumors in mice. J. Clin. Investig..

[132] Soltantoyeh T., Akbari B., Karimi A., Mahmoodi Chalbatani G., Ghahri-Saremi N., Hadjati J., Hamblin M.R., Mirzaei H.R. (2021). Chimeric Antigen Receptor (CAR) T Cell Therapy for Metastatic Melanoma: Challenges and Road Ahead. Cells.

[133] Kang K., Xie F., Mao J., Bai Y., Wang X. (2020). Significance of Tumor Mutation Burden in Immune Infiltration and Prognosis in Cutaneous Melanoma. Front. Oncol..

[134] Baulu E., Gardet C., Chuvin N., Depil S. (2023). TCR-engineered T cell therapy in solid tumors: State of the art and perspectives. Sci. Adv..

[135] Edeline J., Houot R., Marabelle A., Alcantara M. (2021). CAR-T cells and BiTEs in solid tumors: Challenges and perspectives. J. Hematol. Oncol..

[136] Huehls A.M., Coupet T.A., Sentman C.L. (2015). Bispecific T-cell engagers for cancer immunotherapy. Immunol. Cell Biol..

[137] Gros A., Robbins P.F., Yao X., Li Y.F., Turcotte S., Tran E., Wunderlich J.R., Mixon A., Farid S., Dudley M.E. (2014). PD-1 identifies the patient-specific CD8+ tumor-reactive repertoire infiltrating human tumors. J. Clin. Investig..

[138] Chow A., Perica K., Klebanoff C.A., Wolchok J.D. (2022). Clinical implications of T cell exhaustion for cancer immunotherapy. Nat. Rev. Clin. Oncol..

[139] Nassief G., Anaeme A., Moussa K., Mansour A.N., Ansstas G. (2024). Recent Advancements in Cell-Based Therapies in Melanoma. Int. J. Mol. Sci..

[140] Wachsmann T.L.A., Meeuwsen M.H., Remst D.F.G., Buchner K., Wouters A.K., Hagedoorn R.S., Falkenburg J.H.F., Heemskerk M.H.M. (2023). Combining BCMA-targeting CAR T cells with TCR-engineered T-cell therapy to prevent immune escape of multiple myeloma. Blood Adv..

[141] Huang Y., Qin Y., He Y., Qiu D., Zheng Y., Wei J., Zhang L., Yang D.H., Li Y. (2024). Advances in molecular targeted drugs in combination with CAR-T cell therapy for hematologic malignancies. Drug Resist. Updates.

[142] Zhu W.M., Middleton M.R. (2023). Combination therapies for the optimisation of Bispecific T-cell Engagers in cancer treatment. Immunother. Adv..

[143] Lv Y., Luo X., Xie Z., Qiu J., Yang J., Deng Y., Long R., Tang G., Zhang C., Zuo J. (2024). Prospects and challenges of CAR-T cell therapy combined with ICIs. Front. Oncol..

